# The novel *carboxylesterase 1* variant c.662A>G may decrease the bioactivation of oseltamivir in humans

**DOI:** 10.1371/journal.pone.0176320

**Published:** 2017-04-24

**Authors:** Jaeseong Oh, SeungHwan Lee, Howard Lee, Joo-Youn Cho, Seo Hyun Yoon, In-Jin Jang, Kyung-Sang Yu, Kyoung Soo Lim

**Affiliations:** 1 Department of Clinical Pharmacology and Therapeutics, Seoul National University College of Medicine and Hospital, Seoul, Republic of Korea; 2 Department of Transdisciplinary Studies, Graduate School of Convergence Science and Technology, Seoul National University, Seoul, Republic of Korea; 3 Department of Clinical Pharmacology and Therapeutics, CHA University School of Medicine and CHA Bundang Medical Center, Seongnam, Republic of Korea; Georgia State University, UNITED STATES

## Abstract

**Background:**

Human carboxylesterase 1 (CES1) is a serine esterase that hydrolyses various exogenous and endogenous compounds including oseltamivir, a prodrug used to treat influenza. A novel *CES1* c.662A>G single nucleotide polymorphism (SNP) was predicted to decrease CES1 enzymatic activity in an *in silico* analysis. This study evaluated the effect of the c.662A>G SNP on the pharmacokinetics (PK) of oseltamivir in humans.

**Methods:**

A single oral dose of oseltamivir at 75 mg was administered to 20 healthy subjects, 8 heterozygous c.662A>G carriers (c.662AG) and 12 non-carriers (c.662AA). The concentrations of oseltamivir and its active metabolite, oseltamivir carboxylate, were measured in plasma and urine using a validated liquid chromatography-tandem mass spectrometry (LC-MS/MS) method. The PK parameters were calculated using a noncompartmental method. The geometric mean ratios (GMR, c.662AG to c.662AA) of the PK parameters and their 90% confidence intervals (CI) were calculated.

**Results:**

The systemic exposure to oseltamivir, as assessed by the AUC_0-48h_ of oseltamivir, was increased by 10% in c.662AG subjects, whereas the AUC_0-48h_ of oseltamivir carboxylate was 5% lower in c.662AG subjects. The GMR and 90% CI of the metabolic ratio (AUC_0-48h, Oseltamivir carboxylate_/AUC_0-48h, Oseltamivir_) was 0.87 (0.66–1.14). The amount of unchanged oseltamivir excreted in the urine was increased by 15% in subjects with the c.662AG genotype.

**Conclusions:**

This result suggests that CES1 enzymatic activity may be decreased in these heterozygous allele carriers, although further studies are warranted to investigate the clinical implications of this genetic variation on CES1 substrate drugs.

**Trial registration:**

ClinicalTtrials.gov NCT01902342

## Introduction

Oseltamivir is one of the most commonly used antiviral agents to treat and prevent influenza [1–3]. It is an ethyl ester prodrug of the active oseltamivir carboxylate, which selectively inhibits the neuraminidase enzyme of the influenza virus [1–4]. Up to 80% of an orally administered dose of oseltamivir is converted into oseltamivir carboxylate, as mediated by human carboxylesterase 1 (CES1). Oseltamivir carboxylate is eliminated by urinary excretion (S1 Fig) [2, 4]. Other drug metabolizing enzymes (i.e., cytochrome P450 or glucuronosyltransferases) are not involved in the elimination process of oseltamivir or its active metabolite [2, 4].

The human CES enzyme is present as 2 main isozymes, i.e., CES1 and CES2, and the level of expression of these CES isozymes differs among organs. Namely, the CES1 isozyme is highly expressed in the liver, whereas the expression of the CES2 enzyme is high in the intestine [5, 6]. Numerous ester prodrugs (e.g., oseltamivir, clopidogrel, and angiotensin converting enzyme inhibitors), methylphenidate, and some illegal psychotropic drugs (e.g., cocaine and heroin) are substrates for the CES1 enzyme [7–12].

Genetic polymorphisms of the human CES1 enzyme contribute to the large inter-individual variability of its substrate drugs. In earlier *in vitro* and *in vivo* studies, CES1 genetic variants such as c.428G>A (p.Gly143Glu, rs121912777) and c.780delT (p.Asp260fs, rs71647872) were associated with decreased activity of the CES1 enzyme, resulting in altered pharmacokinetic characteristics of methylphenidate, enalapril, clopidogrel and oseltamivir in humans [13–17]. However, those CES1 genetic variants have not been observed in Asians and they are also rarely found in white, black and Hispanic populations [13, 18]. In Asian populations, the genetic polymorphism of CES1 enzyme has been evaluated in Japanese subjects, but the study results based on different study designs and substrates were inconsistent [19, 20]. To identify novel CES1 genetic variants that can alter the CES1 enzyme activity in Asian population, we identified 41 single nucleotide polymorphisms (SNPs) including 14 nonsynonymous variants for CES1 in 200 Koreans [18]. Among them, 3 SNPs (i.e., c.662A>G, rs200707504; c56G>T, rs3826190; c.808G>T, rs115629050) were predicted to decrease CES1 enzymatic activity based on an *in silico* analysis using the PolyPhen-2 software (http://genetics.bwh.harvard.edu/pph2/), and their minor allele frequencies (MAFs) in Koreans were 2%, 1.5% and 0.8%, respectively, compared with 4.57% in the global population [18, 21, 22]. Whereas neither c56G>T nor c.808G>T was associated with a significant effect on CES1-mediated hydrolysis in a previous *in vitro* study [9], the effect of the c.662A>G SNP on the CES1 enzyme has not been characterized previously.

Many people may be exposed to oseltamivir during an influenza pandemic, and the c.662A>G SNP can be an important clinical biomarker if it significantly decreases CES1 enzyme function in humans, although the frequency of the c.662A>G SNP is relatively infrequent. Based on this understanding, we hypothesized that the *CES1* c.662A>G SNP decreases the enzymatic activity of human CES1, thereby decreasing the bioactivation of oseltamivir. To test this hypothesis, the pharmacokinetics (PK) of oseltamivir and its active metabolite, oseltamivir carboxylate, were compared among 20 healthy male volunteers, classified into 2 genotype groups according to their c.662A>G variant status.

## Materials and methods

### Clinical study design

To identify subjects with the c.662A>G SNP, a genotype test was performed using banked blood samples (N = 546) at Seoul National University. After identifying the *CES1* genotype, the subjects who were willing to be enrolled in this study underwent a screening test. Twenty healthy male volunteers were enrolled into this parallel-group clinical study based on their c.662A>G SNP status (8 heterozygous c.662A>G carriers (c.662AG) and 12 non-carriers (c.662AA)). Subjects were admitted to the Clinical Trials Center at Seoul National University Hospital one day before the administration of oseltamivir. After an overnight fast, all the subjects received 75 mg of oseltamivir (Tamiflu^®^ Capsule; Roche Registration Ltd., Welwin Garden City, United Kingdom) with 240 mL of water. The study drug was administered by the investigators. Serial plasma samples were collected at 0 (i.e., pre-dose), 0.5, 1, 1.5, 2, 3, 4, 5, 6, 8, 10, 12, 24, 36 and 48 h post-dose for the analysis of oseltamivir and oseltamivir carboxylate concentrations using a heparinized tube. Urine samples were also collected up to 48 h post-dose. The study subjects participated starting 10 to 12 days from the time of drug administration to the end of the study visit.

The study protocol was approved by the Institutional Review Board of the Seoul National University Hospital, Seoul, Korea, and the study was conducted in accordance with the principles of the Declaration of Helsinki and ICH Good Clinical Practice (clinicaltrials.gov identification number: NCT01902342). Written consent was obtained from all the subjects before any study-related procedure was performed.

### Determination of the oseltamivir and oseltamivir carboxylate concentration

The oseltamivir and oseltamivir carboxylate concentrations in the plasma and urine were determined using a highly specific and sensitive method of liquid chromatography–tandem mass spectrometry (LC-MS/MS) (Agilent 6490 Triple Quadrupole, Agilent Technologies, Santa Clara, CA, USA). To prepare the samples for analysis, an aliquot of the plasma or urine specimen was mixed with acetonitrile in the presence or absence of the internal standard oseltamivir carboxylate-d3. The mixture was vortexed for 30 sec and then centrifuged for 10 min at 14,000 rpm. An aliquot of the supernatant was transferred to an autosampler vial, and 2 μL was injected onto the Kinetex HILIC column (50 mm × 2.1 mm, 5 μm; Phenomenex, Torrance, CA, USA) within a 3 min run at a flow rate of 0.3 mL/min using gradient elution. Mobile phase A consisted of 10 mM ammonium acetate in water, and mobile phase B consisted of 100% acetonitrile. Oseltamivir and oseltamivir carboxylate were quantitatively detected using positive ionization of triple-quadrupole mass spectrometry equipped with electrospray ionization. The method was validated within a ranges of 0.5–100 ng/mL and 20–20,000 ng/mL for oseltamivir, and 2–500 ng/mL and 500–100,000 ng/mL for oseltamivir carboxylate in plasma and urine, respectively.

### Genotyping of *CES1* c.662A>G SNP

Genomic DNA samples were extracted using a QIAamp DNA Mini Kit (QIAgen, Hilden Germany). Target gene-specific primer pairs (CES1-1F: *ctgtggtcctgaaggtcctg*; CES1-1R: *caaccaagctggaagaggag*) and Dr. MAX DNA Polymerase (Doctor Protein INC, Seoul, Korea) were used for the PCR reactions. The PCR amplification conditions consisted of 94°C for 5 min; 35 cycles of 94°C for 30 sec, variable temperature for 30 sec, and 72°C for 40 sec; and finally 72°C for 7 min. PCR products were purified using Millipore plate MSNU030 (Millipore SAS, Molsheim, France). The purified PCR products were then Sanger-sequenced with the BigDye Terminator Sequencing Kit v3.1 on an ABI PRISM 3730xl automated sequencer (Applied Biosystems, Foster City, CA, USA). Nucleotide sequences were determined for both strands of the PCR amplification products at the Macrogen Sequencing Facility (Macrogen Inc., Seoul, Korea). The *CES1* SNP genotyping was focused on c.662A>G and did not cover other *CES1* SNPs.

### Pharmacokinetics data analysis

The plasma concentrations of oseltamivir and oseltamivir carboxylate were analysed by noncompartmental analysis using Phoenix^®^ WinNonlin^®^ software version 1.3 (Certara, St. Louis, MO, USA). The area under the concentration-time curve from time 0 to 48 h post-dose of oseltamivir (AUC_0-48h, oseltamivir_) and oseltamivir carboxylate (AUC_0-48h, oseltamivir carboxylate_) was calculated using the linear up, log down trapezoidal method [23]. The metabolic ratio was calculated as AUC_0-48h, oseltamivir carboxylate_ / AUC_0-48h, oseltamivir_. The observed concentrations and times were used to estimate the maximum concentration (C_max_) and time to reach the C_max_ (T_max_) for oseltamivir and oseltamivir carboxylate. The apparent terminal elimination rate constant (λ_z_) was estimated from a regression of log-transformed plasma concentrations of oseltamivir and oseltamivir carboxylate versus time over the terminal log-linear disposition portion of the concentration-time profiles, and the elimination half-life (t_1/2_) was calculated as the natural logarithm of 2 divided by λ_z_. Total apparent clearance (CL/F) of oseltamivir was calculated as the administered dose (75 mg) over the AUC_0-48h, oseltamivir,_ and the apparent volume of distribution was calculated as the CL/F divided by λ_z_. The total urinary excreted amount of oseltamivir and oseltamivir carboxylate during 48 h (Ae_0-48h_) was calculated by multiplying the volume of excreted urine and the urinary concentration of oseltamivir and oseltamivir carboxylate, respectively.

### Statistical analysis

The sample size was estimated to detect a 25% difference in the metabolic ratio (equivalent to a mean difference of 0.29 in the log-transformed metabolic ratio) of oseltamivir between the two genotype groups, with an 80% power and a 5% significance level using a two-sided T-test. The total coefficient of variation value of the metabolic ratio in wild-type subjects was assumed to be 20% (equivalent to a σ of 0.20 in the log-transformed metabolic ratio) according to an earlier study [15]. A total number of 18 subjects was sufficient to reach 80% power for showing a minimal detectable difference of 1.45 (mean difference/σ) in the log-transformed metabolic ratio with a 5% significance level using a two-sided T-test. All the demographic characteristics and PK parameters are presented as arithmetic mean and standard deviation (SD). A general linear model was developed to estimate the geometric mean ratios (GMRs) of the PK parameters and their 90% confidence intervals (CIs) for heterozygous carriers (c.662AG) over non-carriers (c.662AA). Log-transformed PK parameters were used as dependent variables in the model, where genotype was used as a fixed effects. Two one-sided T-tests at the 5% level of significance were conducted to determine the 90% CI around the ratio of the log-transformed population means for PK parameters. The Wilcoxon two-sample test was performed to identify significant differences in the demographic characteristics between the genotype groups. A *p*-value <0.05 was considered statistically significant. Statistical analyses were performed using SAS software version 9.3 (SAS Institute Inc., Cary, NC, USA).

## Results

### Frequency of the *CES1* c.662A>G SNP

**Fig 1:**
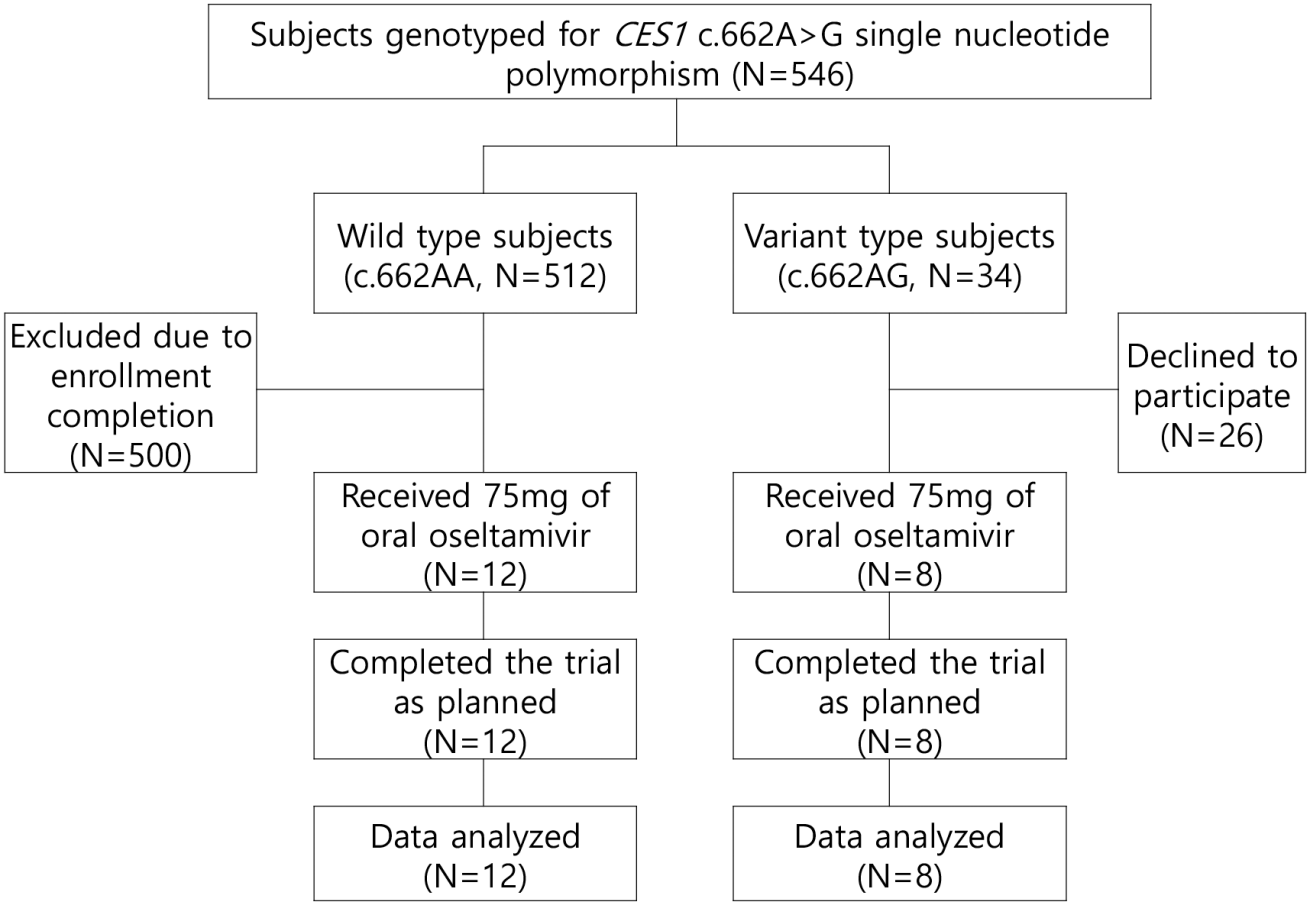
Study flowchart.

A total of 546 subjects were genotyped, and 512 and 34 of these had the c.662AA and c.662AG genotypes, respectively (Fig 1). None had the c.662GG genotype, leading to a MAF of 3.1%. The MAF observed in this study was compatible with that obtained in our earlier study but was higher than the value observed in the dbSNP (0.1%, http://www.ncbi.nlm.nih.gov/SNP) [18].

### Demographic characteristics

A total of 20 healthy Korean male subjects were enrolled from July 2013, and all the subjects completed the trial as planned until December 2013. Twelve and eight subjects had the c.662AA and c.662AG genotype, respectively (Fig 1). The demographic characteristics were similar between the genotype groups, at 28 vs. 27 years (age), 1.76 vs. 1.74 m (height), 72.8 vs. 67.5 kg (body weight), and 23.5 vs. 22.2 kg/m^2^ (BMI) for the c.662AA and c.662AG genotype, respectively (*p* > 0.05 for all demographic characteristics).

### Effect of the *CES1* c.662A>G SNP on the PK of oseltamivir

**Fig 2:**
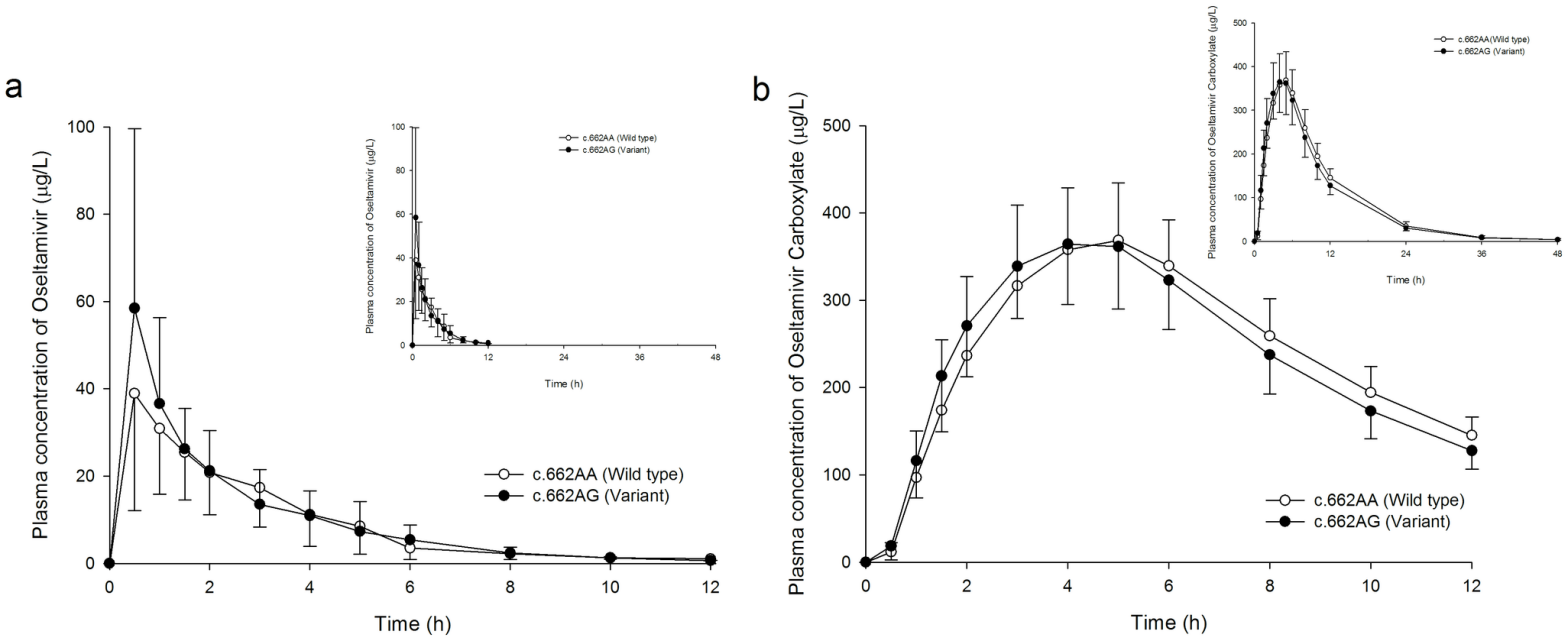
Mean plasma concentration profiles of oseltamivir and oseltamivir carboxylate after a single oral administration of oseltamivir at 75 mg. The open and filled circles denote the mean values in subjects with the c.662AA and c.662AG genotype, respectively. The error bars represent the standard deviation.

**Fig 3:**
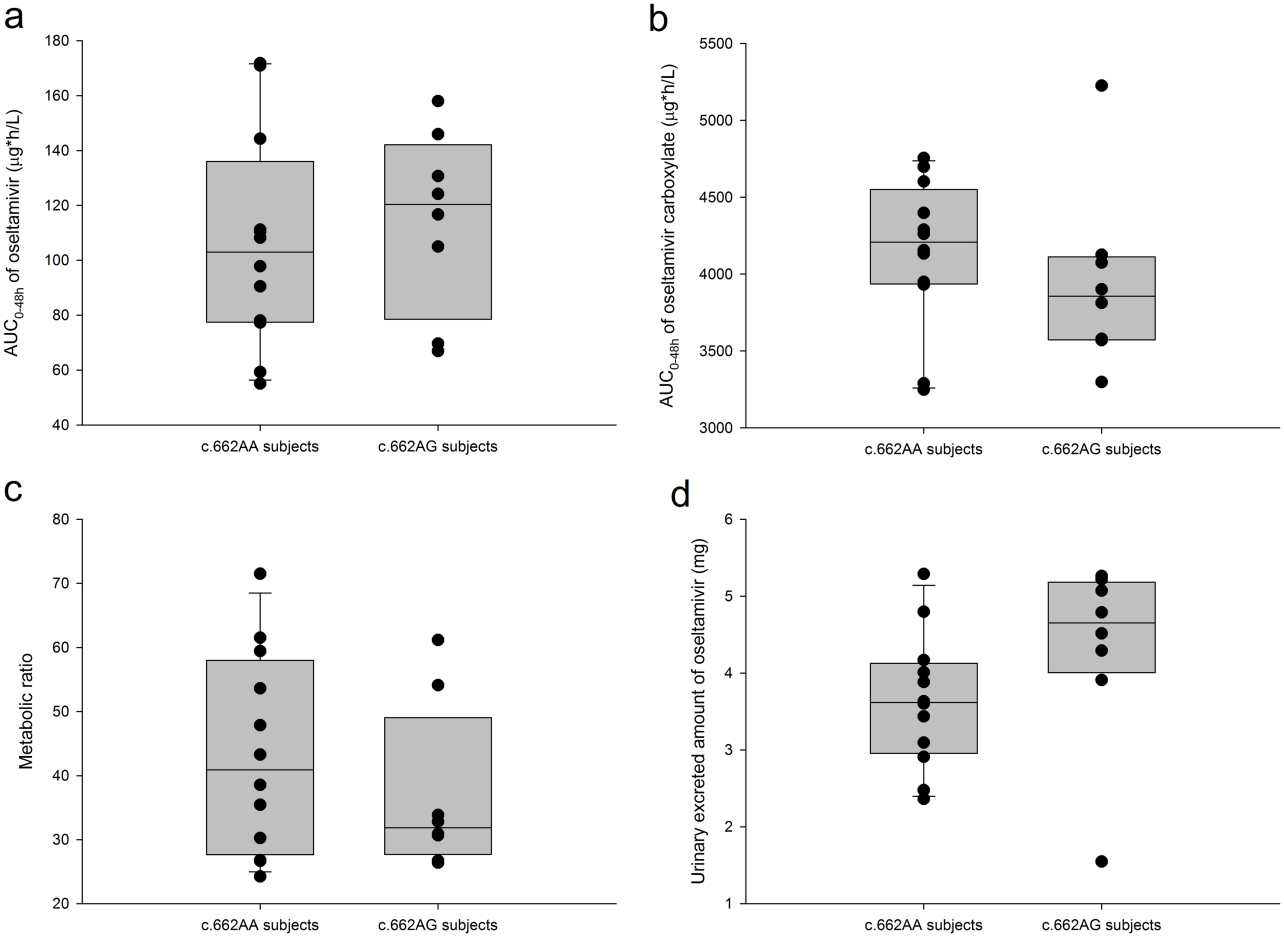
Comparison of PK parameters between genotype groups. The metabolic ratio was calculated as the AUC_0-48h, oseltamivir carboxylate_ / AUC_0-48h, oseltamivir._ The solid lines across the box, the top edge, and the bottom edge represent the median, the 75^th^ percentile, and the 25^th^ percentile, respectively. The horizontal lines connected with the whiskers extending from the box denote the 90^th^ and 10^th^ percentiles, respectively. The dots outside of the whiskers represent outliers.

The mean metabolic ratio of oseltamivir was approximately 13% lower in subjects with the c.662AG genotype than in subjects with the c.662AA genotype, although this effect failed to reach statistical significance (Fig 2, Table 1). The GMR (90% CI) for the metabolic ratio and the AUC_0-48h_ of oseltamivir and oseltamivir carboxylate were 0.87 (0.66–1.14), 1.10 (0.83–1.45) and 0.95 (0.86–1.05), respectively. The CL/F and Vz/F of oseltamivir were not significantly lower in subjects with the c.662AG genotype, and the GMR (90% CI) for the corresponding parameters was 0.91 (0.70–1.20) and 0.84 (0.62–1.15), respectively (Fig 3, Table 1). The amount of oseltamivir excreted in the urine was not significantly greater in subjects with the c.662AG genotype than in those with the c.662AA genotype, and the GMR (90% CI) for the Ae_0-48_ of oseltamivir was 1.15 (0.9–1.48) (Fig 3, Table 1).

**Table 1:** Pharmacokinetic characteristics of oseltamivir in CES1 c.662A>G and wild-type subjects.

			Subject group		Geometric mean ratio (Variant subjects/wild-type subjects) (90% CI)	P-value
			Variant subjects (c.662AG) (N = 8)	Wild-type subjects (c.662AA) (N = 12)		
**Demographic Characteristics**			26.6 ± 3.2	28.3 ± 4.1	.	0.362
			1.74 ± 0.03	1.76 ± 0.04	.	0.471
			67.5 ± 6.2	72.8 ± 6.4	.	0.114
			22.2 ± 1.7	23.5 ± 1.6	.	0.114
**Pharmacokinetic parameter**	**Oseltamivir**	**Metabolic Ratio**^1^	37.1 ± 13.1	43.3 ± 15.6	0.87 (0.66–1.14)	0.386
		**AUC**_**0-48h**_ **(μg∙h/L)**	114.6 ± 33.0	106.2 ± 39.0	1.10 (0.83–1.45)	0.555
		**C**_**max**_ **(μg/L)**	68.41 ± 35.3	47.5 ± 21.7	1.42 (0.98–2.06)	0.122
		**T**_**max**_ **(h)**	0.5 (0.5–1.5)	0.5 (0.5–2.0)	.	0.675
		**CL/F (L/h)**	704.1 ± 242.8	779.5 ± 281.3	0.91 (0.70–1.20)	0.581
		**Vz/F (L)**	1115.6 ± 226.6	1449.5 ± 737.8	0.84 (0.62–1.15)	0.355
		**t**_**1/2**_ **(h)**	1.2 ± 0.5	1.3 ± 0.5	0.92 (0.67–1.26)	0.657
		**Ae**_**0-48h**_ **(mg)**	4.33 ± 1.22	3.64 ± 0.87	1.15 (0.9–1.48)	0.334
	**Oseltamivir carboxylate**	**AUC**_**0-48h**_ **(μg∙h/L)**	3948.1 ± 586.0	4142.1 ± 486.8	0.95 (0.86–1.05)	0.410
		**C**_**max**_ **(μg/L)**	374.7 ± 65.7	377.9 ± 70.9	1.00 (0.85–1.17)	0.967
		**T**_**max**_ **(h)**	3.0 (5.0–6.1)	3.0 (5.0–6.0)	.	1.0
		**t**_**1/2**_ **(h)**	6.5 ± 0.3	6.8 ± 1.2	0.96 (0.86–1.07)	0.507
		**Ae**_**0-48h**_ **(mg)**	59.95 ± 9.53	61.83 ± 5.41	0.96 (0.86–1.07)	0.513

AUC_0-48h,_ C_max_, CL/F, V_Z_/F_,_ t_1/2_, AE_0-48h_ and CL_R_ are presented as the arithmetic means ± standard deviations.

T_max_ is presented as the median (min—max).

^1^ The metabolic ratio was calculated as AUC_0-48h, oseltamivir carboxylate_ / AUC_0-48h, oseltamivir._

## Discussion

In this study, the metabolic ratio, apparent clearance and apparent volume of distribution for oseltamivir were 10–16% lower in subjects with the c.662AG genotype (i.e., heterozygous carriers) than those with the c.662AA genotype (i.e., wild-types) for the CES1 enzyme (Table 1, Fig 3), although we failed to show the statistical significance of these differences. The c.662A>G SNP is associated with a missense mutation in the 5^th^ exon of the *CES1* gene (reference sequence NG_012057.1) that leads to a glutamate (Glu)-to-glycine (Gly) amino acid change at position 220 [18]. Glu220 is located next to Ser221, one of the three catalytic residues of the CES1 enzyme, and the switch of this amino acid with glycine at position 220 has been suggested to decrease the catalytic function of the CES1 enzyme [21, 22, 24–26]. The intrinsic clearance of oseltamivir by the human CES1 enzyme is rapid even in neonates and infants, and the overall clearance of oseltamivir to oseltamivir carboxylate approached the hepatic blood flow (i.e., the extraction ratio of oseltamivir in the liver is close to 1 in humans) [27]. For high extraction ratio drugs such as oseltamivir and propranolol, a slight difference in bioavailability (F) may lead to a large variability in drug exposures [28, 29]. Therefore, we postulate that the decreased CES1 enzymatic activity in heterozygous carriers (i.e., c.662A>G SNP), as evidenced by a 13% lower metabolic ratio than that of wild-type subjects in the present study, resulted in an increased bioavailability of oseltamivir (or a decreased pre-systemic clearance), leading to a 10% greater systemic exposure to oseltamivir and a 42% higher maximum concentration (Table 1, Fig 3).

To treat influenza, oseltamivir is administered for 5 days, although this treatment can be extended for the prevention of influenza. Oseltamivir and oseltamivir carboxylate show a linear PK characteristic profile in the clinical dose range, and their plasma concentrations at steady state can be adequately predicted using the PK parameters estimated after a single administration [2, 4]. Therefore, individuals who carry the *CES1* c.662A>G SNP are likely to have an increased systemic exposure to oseltamivir at a steady state, whereas the systemic exposure to its active metabolite, oseltamivir carboxylate, is slightly lower.

The PK characteristics of oseltamivir that we observed in carriers of the *CES1* c.662A>G SNP may have important implications for the efficacy and safety profiles of this drug. First, the plasma concentration of oseltamivir carboxylate has been shown to be associated with the anti-viral effect of oseltamivir [4]. The metabolic ratio in *CES1* c.428G>A heterozygous carriers was only approximately 16% lower than that in wild-type subjects, whereas homozygous carriers showed a 23% lower metabolic ratio than wild-type subjects [15], which is comparable to the effects found in the present study (13% lower, Table 1). Although no c.662A>G homozygous carriers were enrolled in the present study, their bioactivation of oseltamivir could have been much lower than those of both heterozygous carriers and wild-type subjects. Therefore, the antiviral efficacy of oseltamivir could be much more compromised in homozygous carriers as a consequence of decreased CES1 enzyme activity. Second, abnormal behaviours and even death have been reported after oseltamivir treatment in Japan, which may have been caused by the adverse effects of oseltamivir on the central nervous system (CNS) [30]. Because oseltamivir is more lipophilic than oseltamivir carboxylate, penetration into the CNS was higher for oseltamivir than for its active metabolite [31]. Therefore, the increased systemic exposure to oseltamivir in CES1 variants can lead to increased concentrations in the brain, which might increase the likelihood of CNS toxicity.

Altered CES1 enzyme activity can also influence the PK, pharmacodynamics (PD) and clinical outcomes of various drugs such as cocaine, methylphenidate, enalapril and clopidogrel [7–12, 16, 17]. Inhibition of the CES1 enzyme and its loss-of-function genetic variant (i.e., c.428G>A) have been associated with higher concentrations of clopidogrel’s active metabolite, which enhanced the antiplatelet activity of clopidogrel in *in vitro* and *in vivo* studies [9, 10, 17]. Additionally, subjects with the c.428G>A SNP showed higher methylphenidate concentrations, requiring lower doses of methylphenidate for symptom reduction in patients with attention deficit hyperactivity disorder carrying the variant allele [13, 32]. Therefore, further studies are warranted to investigate the role of the *CES1* c.662A>G SNP on the PK, PD and clinical outcomes of various CES1 substrate drugs.

The present study has several limitations. First, the role of the c.662A>G SNP on CES1 enzyme structure or its catalytic function had not been evaluated in *in vitro* studies before the present *in vivo* study was performed. However, it was expected that the c.662A>G SNP would act as a functional SNP based on *in silico* analysis, and the clinical significance of the c.662A>G SNP remained to be evaluated *in vivo* [18]. Additionally, the study was performed in a relatively small number of subjects due to the low frequencies of the c.662A>G SNP in Koreans. A *post hoc* power analysis showed that the actual statistical power was only 31% for the metabolic ratio, and 25% decrements in the metabolic ratio should have been observed to attain the same statistical power (i.e., 80%) with 20 subjects at the same significance level of 5%. Therefore, the number of subjects was not large enough to detect a possible difference between genotypes. Finally, the results of the present study cannot be extrapolated to a general population due to differences in CES1 enzyme activity among different age groups. For instance, CES1 enzyme activity is lower in populations younger and older than in the young adults we studied, and the difference in catalytic function due to genetic polymorphisms would be smaller in those age groups [33, 34].

In conclusion, the bioactivation of oseltamivir was decreased in subjects with the c.662AG genotype compared to wild-type subjects, although this difference failed to reach statistical significance. This finding suggests that the *CES1* c.662A>G SNP may be associated with lower enzymatic activity of the human CES1 enzyme, which may contribute to inter-individual variability in the effectiveness of drugs metabolized via CES1 activity. Further studies including a larger number of subjects are warranted to investigate the impact of the *CES1* c.662A>G variant on CES1 enzyme activity.

## Supporting information

S1 Fig Disposition of oseltamivir and oseltamivir carboxylate in the human body. (TIF)

S1 File CONSORT checklist. (PDF)

S2 File Study protocol. (DOC)

S3 File Pharmacokinetic dataset. (ZIP)
