# Supplementary figures and images for: The novel carboxylesterase 1 variant c.662A>G may decrease the bioactivation of oseltamivir in humans
Source: PLoS One. 2017 Apr 24;12(4):e0176320. doi: 10.1371/journal.pone.0176320 (PMC5402961; doi:10.1371/journal.pone.0176320)

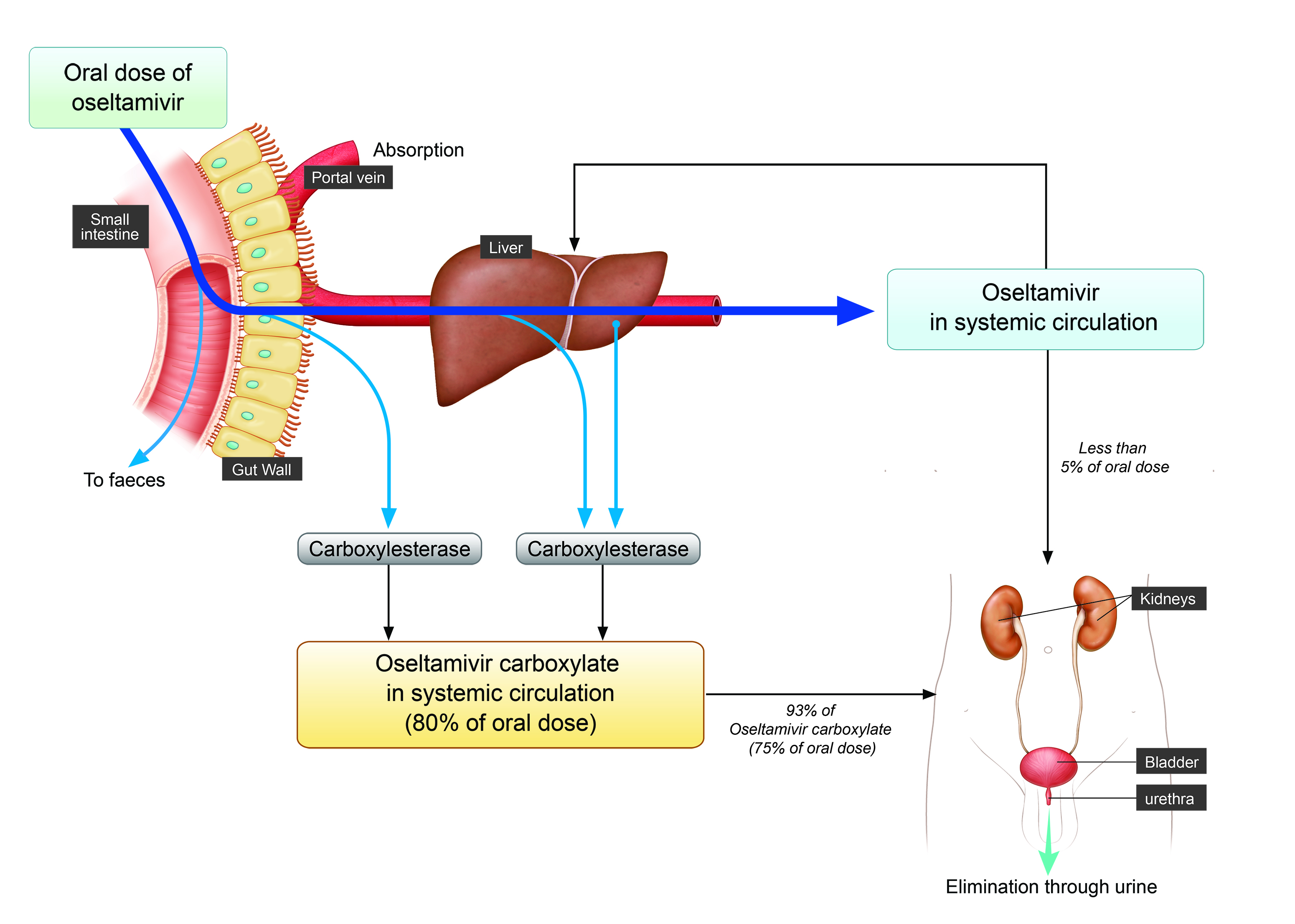

Supplement: S1 Fig — (TIF) [file pone.0176320.s001.tif]
