# Supplementary material for: The novel carboxylesterase 1 variant c.662A>G may decrease the bioactivation of oseltamivir in humans
Source: PLoS One. 2017 Apr 24;12(4):e0176320. doi: 10.1371/journal.pone.0176320 (PMC5402961; doi:10.1371/journal.pone.0176320)
Supplement: S2 File — (DOC) [file pone.0176320.s003.doc]

| 임 상 연 구 계 획 서 (Clinical study Protocol) |
| --- |

**건강 자원자에서 CES1 유전형에 의한 Oseltamivir 의 약동학적 특성을 평가하기 위한 임상연구**

**A clinical trial to investigate the influence of CES1 polymorphism on pharmacokinetic characteristics of oseltamivir in healthy Korean volunteers**

**Version No. 1.3**

**서울대학교 의과대학 임상약리학교실**

**서울대학교병원 임상약리학과
(Department of Clinical Pharmacology and Therapeutics, Seoul National University School of Medicine and Hospital)**

# **임상연구 계획서 요약(summary of study protocol)**

| **제 목(Study title)** | 건강 자원자에서 CES1 유전형에 의한 Oseltamivir 의 약동학적 특성을 평가하기 위한 임상연구 (A clinical trial to investigate the influence of CES1 polymorphism on pharmacokinetic characteristics of oseltamivir in healthy Korean volunteers) |
| --- | --- |
| **임상시험책임자(Principal investigator)** | 서울대학교 의과대학 임상약리학교실/ 서울대학교병원 임상약리학과  (Department of Clinical Pharmacololgy and Therapeutics, Seoul National University School of Medicine and Hospital)  진료교수 임 경 수 MD, PhD (Prof. Kyoung Soo Lim) |
| **임상시험기관(Study center)** | 서울대학교병원 임상시험센터(Clinical trial center, Seoul National University Hospital)  서울특별시 종로구 대학로 101 |
| **연구기간(Study period)** | IRB 승인일 이후 1년 또는 총 20명의 시험대상자(이하 대상자 혹은 피험자)가 임상연구를 완료하는 시점 (1 year after IRB approval or until every subject complete the trial) |
| **임상시험단계(Type of study)** | 기타(학술연구) (other) |
| **목적(Purpose of study)** | 건강 성인을 대상으로 oseltamivir의 bioactivation에 영향을 미칠 것으로 예상되는 CES1의 유전적 변이가 oseltamivir의 약동학에 미치는 영향을 평가한다(To assess the effect of *CES1* genetic variability on the pharmacokinetics of oseltamivir). |
| **연구설계(Study design)** | 공개, 단회투약, 약동학 임상시험 (Open label, Single dose, Pharmacokinetic study) |
| **소요기간(Study period)** | 10 ~ 12 일 (10 to 12 days) |
| **대상자 수(Number of Study subjects)** | 총 20 명 (A total of 20 subject)   | *CES1* 유전형 (CES1 genotype) | 대상자수(Number of subjects) | | --- | --- | | 662A>G | 8 | | Wild type | 12 | |
| **임상시험용의약품(Study drug)** | Oseltamivir 75 mg capsule (Tamiflu®, Roche) |
| **선정제외기준(Inclusion and exclusion criteria)** | 선정기준(Inclusion Criteria)   1. 모든 스크리닝 검사 이전에, 본 임상시험의 특성에 대해 설명을 들은 후, 본   시험의 참여에 자발적으로 동의하고 IRB 승인을 얻은 동의서에 서명한 자 (Agreement with written informed consent)   1. 만 20세 이상 만 45세 이하의 건강한 한국인 남성 혹은 여성 자원자   (Adult healthy male or female subject age 20 to 45)  제외기준(Exclusion Criteria)   1. 유의한 호흡기, 순환기, 신장, 위장관, 간, 내분비, 혈액, 신경 (운동실조 포함), 정신 질환 혹은 기타 만성 질환, 알코올 혹은 약물 중독에 대한 임상적인 증거가 있는 자(Clinically significant, active gastrointestinal system, cardiovascular system, pulmonary system, renal system, endocrine system, blood system, digestive system, central nervous system, mental disease or malignancy 2. 임상연구용 의약품의 투약 14일 이내에 유의한 약물상호작용이 알려진 약물 또는 연구자가 판단하기에 적합하지 않다고 판단되는 약물을 복용한 자(Medication with any drug which may affect the pharmacokinetics of oseltamivir within 14 days) 3. 임상연구 개시 전 30일 이내 혈액을 공여하거나 60일 이내에 다른 임상시험용의약품 혹은 시판 중인 약물의 임상연구에 참여한 자(Previously donate whole blood within 30 days or Previously participated in other trial within 60 days) 4. 임상연구용 의약품에 대한 과민반응 등 유의한 이상반응을 보인 적이 있는 자(Subject with known for hypersensitivity reactions to oseltamivir) 5. 시험기간 중 임신을 계획하고 있거나 계획 중이 아니어도 인정받는 피임법 (예: 본인 및 파트너의 불임수술, 파트너의 자궁 내 피임기구, 간벽 피임법, 격막 또는 콘돔의 병용)을 사용할 수 없는 자(Subject who can not perform contraception during study periods) 6. 임신 또는 수유중인 여성 대상자(Female woman who are pregnant or are breast feeding) 7. 임상실험실검사 결과를 비롯한 기타 사유로 인하여 연구자가 임상연구 참여에 부적합하다고 판단한 자(An impossible one who participates in clinical trial by investigator's decision including laboratory test result) |
| **연구방법(Study method)** | (admission) (Drug administration) (Discharge) (Post-study visit)  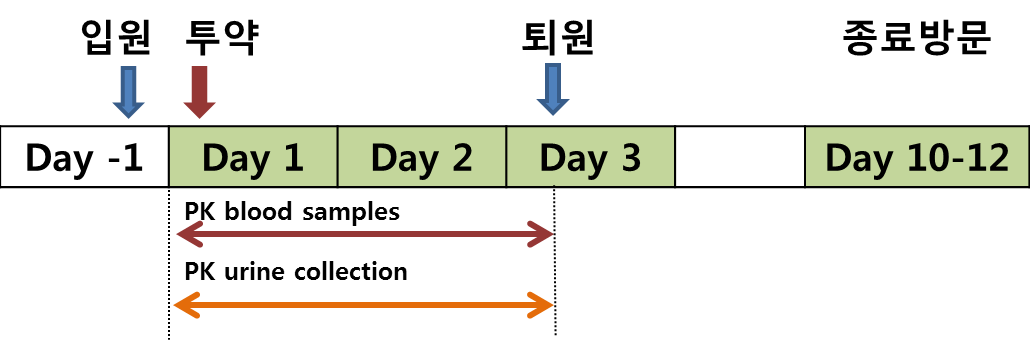  본 임상연구에 참여하고자 하는 건강자원자의 CES1 유전형 정보를 확인한 후, CES1 유전형을 고려하여 자원자를 본 임상연구에 등록한다. 임상연구용의약품 투약 예정일 하루 전(-1d)으로부터 30일 이내(-30d ~ -1d)에 스크리닝 검사를 시행하여, 본 임상연구에 적합하다고 판단되는 대상자를 선정한다.  선정된 대상자는 입원 예정일(-1d) 오후 9시까지 서울대학교병원 임상시험센터에 입원한다. 입원 둘째 날(1d) 오전 9시경 Oseltamivir 75 mg capsule 을 투약한다. 투약 직전 방광을 비운 후 48시간 동안 집뇨를 수행하며 예정된 시간에 약동학 채혈을 수행한다. (8mL씩 15회) 대상자는 48시간 째 약동학 채혈 후 퇴원할 수 있다.  모든 일정을 마친 대상자는 10d ~ 12d 중 하루에 post-study visit을 한다. (Subjects will admit to the Clinical Trials Center at Seoul National University Hospital one day before the administration of oseltamivir. After an overnight fast, all the subjects will receive 75 mg of oseltamivir with 240 mL of water by the investigator. Serial plasma samples will be collected at 0 (ie, pre-dose), 0.5, 1, 1.5, 2, 3, 4, 5, 6, 8, 10, 12, 24, 36 and 48 h post-dose for the analysis of oseltamivir and oseltamivir carboxylate concentrations using a heparinized tube. Urine samples will also be collected up to 48 h post-dose. Subjects will perform post-study visit during day 10 – 12.) |
| **평가기준(Endpoints)** | - 약동학적 평가(Pharmacokinetic assessment) - 평가 변수(Pharmacokinetic endpoints)   - Oseltamivir 및 active metabolite (oseltamivir carboxylate)의 C_max_, AUC_last_, AUC_inf_, CL/F, CL_R_/F, t_1/2_, metabolic ratio 등 (C_max_, AUC_last_, AUC_inf_, CL/F, CL_R_/F, t_1/2_, metabolic ratio of oseltamivir and its active metabolite) - 안전성 및 내약성 평가(Tolerability assessment) - 이상반응, 신체검진, 활력징후, 심전도, 임상실험실 검사 (Adverse event, physical exam, vital sign, ECG, laboratory test) |
| **자료분석 및 통계분석 방법(Data analysis)** | - 약동학 - Oseltamivir 및 active metabolite (oseltamivir carboxylate)의 약동학 파라미터(C_max_, AUC_last_, AUC_inf_, CL/F, CL_R_/F, t_1/2_, metabolic ratio)는 비구획방법을 이용하여 산출한다. - 약동학적 파라미터들을 유전형별로 기술통계학적으로 분석한다. - Wild type에 대한 662A>G 군의 약동학 파라미터들(C_max_, AUC_last_, CL/F, metabolic ratio)에 대한 점추정치를 구하고 유의수준 0.05에서 이의 90% 신뢰구간을 산출한다. - 필요시 기타 적합한 통계분석 방법을 이용하여 분석할 수 있다. - 안전성 / 내약성 - 필요시 정량적인 안전성 및 내약성 자료에 대해 기술통계학적으로 제시할 수 있다. - 병력과 이상반응은 MedDRA (버전 12.0 또는 사용 가능한 최신 버전) 용어로 기술한다.   (The plasma concentrations of oseltamivir and oseltamivir carboxylate are analysed by noncompartmental analysis using Phoenix® WinNonlin® software version 1.3 (Certara, St. Louis, MO, USA). All the demographic characteristics and PK parameters will be presented as arithmetic mean and standard deviation (SD). A general linear model was developed to estimate the geometric mean ratios (GMRs) of the PK parameters and their 90% confidence intervals (CIs) for heterozygous carriers (c.662AG) over non-carriers (c.662AA). The Wilcoxon two-sample test will be performed to identify significant differences in the demographic characteristics between the genotype groups. A p-value <0.05 is considered statistically significant. Statistical analyses will be performed using SAS software version 9.3 (SAS Institute Inc., Cary, NC, USA).) |

# **연구일정요약(Summary of study procedure)**

| **Period** | **Day** | **Planned**  **time** | **Time** | **Event** | **Genotype test[[1]](#footnote-2)** | **PK[[2]](#footnote-3)** | **Drug administration** | **Urine collection[[3]](#footnote-4)** | **Lab test[[4]](#footnote-5)** | **ECG[[5]](#footnote-6)** | **Vital**  **sign[[6]](#footnote-7)** | **Physical exam[[7]](#footnote-8)** |
| --- | --- | --- | --- | --- | --- | --- | --- | --- | --- | --- | --- | --- |
| Genotype test |  |  |  |  | X |  |  |  |  |  |  |  |
| Screening | -30D ~ -1D |  |  | Outpatient visit |  |  |  |  | X | X | X | X |
| Admission | -1D | -12h | 21:00 | Admission |  |  |  |  |  |  |  |  |
|  | 1D | 0h | 9:00 | Drug administration |  | X | X |  |  |  | X | X |
|  |  | 0.5h | 9:30 |  |  | X |  | 0-4h |  |  |  |  |
|  |  | 1h | 10:00 |  |  | X |  |  |  |  |  |  |
|  |  | 1.5h | 10:30 |  |  | X |  |  |  |  |  |  |
|  |  | 2h | 11:00 |  |  | X |  |  |  |  |  |  |
|  |  | 3h | 12:00 |  |  | X |  |  |  |  | X |  |
|  |  | 4h | 13:00 | Lunch |  | X |  |  |  |  |  |  |
|  |  | 5h | 14:00 |  |  | X |  | 4-8h |  |  | X |  |
|  |  | 6h | 15:00 |  |  | X |  |  |  |  |  |  |
|  |  | 8h | 17:00 |  |  | X |  |  |  |  |  |  |
|  |  | 10h | 19:00 | Dinner |  | X |  | 8-12h |  |  |  |  |
|  |  | 12h | 21:00 |  |  | X |  |  |  |  |  |  |
|  | 2D | 24h | 9:00 |  |  | X |  | 12-24h |  |  |  |  |
|  |  | 36h | 21:00 |  |  | X |  | 24-48h |  |  |  |  |
| Discharge | 3D | 48h | 9:00 | Discharge |  | X |  |  |  |  | X |  |
| Post  study  visit (PSV) | 10D ~ 12D | 0h | 9:00 | Outpatient visit |  |  |  |  | X |  | X |  |

목 차(Table of contents)

[**임상연구 계획서 요약(summary of study protocol)** 2](#__RefHeading___Toc441155422)

[**연구일정요약(Summary of study procedure)** 6](#__RefHeading___Toc441155423)

[목 차(Table of contents) 1](#__RefHeading___Toc441155424)

[**1.** **임상시험실시기관명 및 주소(Name of study center and address)** 4](#__RefHeading___Toc441155425)

[**2.** **임상시험책임자, 담당자 및 공동 연구자의 성명 및 직명(Name investigators)** 4](#__RefHeading___Toc441155426)

[2.1. 임상시험책임자(Principal Investigator) 4](#__RefHeading___Toc441155427)

[2.2. 공동연구자(Co-investigator) 4](#__RefHeading___Toc441155428)

[2.3. 임상시험 담당자(Sub-investigators) 4](#__RefHeading___Toc441155429)

[2.4. 관리약사(Name of pharmacists) 4](#__RefHeading___Toc441155430)

[**3.** **임상연구의 목적 및 배경(Background and purpose of study)** 5](#__RefHeading___Toc441155431)

[3.1. 배경(Background) 5](#__RefHeading___Toc441155432)

[3.2. 연구목적(Purpose of study) 7](#__RefHeading___Toc441155433)

[**4.** **윤리적 고려사항(Ethical consideration)** 7](#__RefHeading___Toc441155434)

[**5.** **임상연구용 의약품(Study drug)** 8](#__RefHeading___Toc441155435)

[5.1. 시험약 Oseltamivir 8](#__RefHeading___Toc441155436)

[5.2. 적응증(Indication) 8](#__RefHeading___Toc441155437)

[5.3. 용법, 용량, 예측 이상반응(부작용), 상호작용 및 사용상의 주의사항(Detailed information) 8](#__RefHeading___Toc441155438)

[5.4. 임상연구용의약품의 공급 및 라벨링(Labeling of study drug) 8](#__RefHeading___Toc441155439)

[**6.** **대상자 선정기준 및 대상자 수 산출 근거(Inclusion and exclusion criteria and determination of subject number)** 9](#__RefHeading___Toc441155440)

[6.1. 선정기준(Inclusion criteria) 9](#__RefHeading___Toc441155441)

[6.2. 제외기준(Exclusion criteria) 9](#__RefHeading___Toc441155442)

[6.3. 대상자 수(Determination of subject number) 10](#__RefHeading___Toc441155443)

[**7.** **대상자의 관리(Restrictions to subjects)** 10](#__RefHeading___Toc441155444)

[**8.** **임상연구 기간(Study periods)** 11](#__RefHeading___Toc441155445)

[**9.** **임상연구 방법(Study method)** 11](#__RefHeading___Toc441155446)

[9.1. 대상자 군 및 개요(Subject group) 11](#__RefHeading___Toc441155447)

[9.2. 투약 방법 및 임상시험(Study drug admistration and study method) 11](#__RefHeading___Toc441155448)

[9.3. 채혈방법, 채혈량, 집뇨량 및 검체보관(Blood and urine sampling method, volume of blood and urine, sample handling method) 12](#__RefHeading___Toc441155449)

[9.4. 순응도 평가(Assessment of compliance) 12](#__RefHeading___Toc441155450)

[9.5. 채혈 및 집뇨 시간의 설정(Collection time for blood and urine samples) 13](#__RefHeading___Toc441155451)

[9.6. 대상자 SN 및 AN 부여 방법(Allocation of subject number) 13](#__RefHeading___Toc441155452)

[9.7. 검사 관찰 시점 및 항목(Assessment time and items) 14](#__RefHeading___Toc441155453)

[**9.7.1.** **약물유전학 검사(Pharmacogenetic test)** 14](#__RefHeading___Toc441155454)

[**9.7.2.** **스크리닝(Screening) 검사(D-30~D-1)(Screening visit. D-30 to D-1)** 14](#__RefHeading___Toc441155455)

[**9.7.3.** **투약 전/후(Before and after drug administration)** 15](#__RefHeading___Toc441155456)

[**9.7.4.** **종료방문 검사(Post-study visit)** 15](#__RefHeading___Toc441155457)

[**10.** **대상자의 대체 및 탈락 기준(Subject dropout and replacement)** 15](#__RefHeading___Toc441155458)

[10.1. 분석군(Full Analysis set) 15](#__RefHeading___Toc441155459)

[10.2. 대상자의 대체(Subject replacement) 15](#__RefHeading___Toc441155460)

[10.3. 대상자의 탈락 기준(Dropout criteria) 15](#__RefHeading___Toc441155461)

[**11.** **대상자의 안전 보호 및 이상반응 발생 등에 대한 대책** 16](#__RefHeading___Toc441155462)

[**12.** **평가방법(Endpoints)** 17](#__RefHeading___Toc441155463)

[12.1. 약동학 평가(Pharmacokinetic assessment) 17](#__RefHeading___Toc441155464)

[12.2. 통계분석(Statistical analysis) 17](#__RefHeading___Toc441155465)

[12.3. 안전성 평가(Safety assessment) 18](#__RefHeading___Toc441155466)

[**13.** **이상반응(Adverse event, AE)** 18](#__RefHeading___Toc441155467)

[13.1. 이상반응(Adverse Event, AE)의 정의(Definition of AE) 18](#__RefHeading___Toc441155468)

[13.2. 이상약물반응(Adverse Drug Reaction, ADR)의 정의(Definition of adverse drug reaction) 18](#__RefHeading___Toc441155469)

[13.4. 이상반응의 기록(Recording of adverse event) 19](#__RefHeading___Toc441155470)

[13.5. 이상반응의 중증도 및 시험약과의 인과관계 평가(Assessment method for severity and relationship of AE with IP) 20](#__RefHeading___Toc441155471)

[**13.5.1.** **이상반응의 중증도 평가(Assessment method for severity of AE)** 20](#__RefHeading___Toc441155472)

[**13.5.2.** **이상반응의 시험약과의 인과관계 평가(Assessment method for relationship of AE with IP)** 20](#__RefHeading___Toc441155473)

[**13.5.3.** **이상반응의 추적 관찰(Follow up of AE)** 21](#__RefHeading___Toc441155474)

[13.6. 중대한 이상반응의 보고(Reporting serious AE) 21](#__RefHeading___Toc441155475)

[**14.** **자료 처리 및 자료의 질 보장(Data management and quality assurance)** 21](#__RefHeading___Toc441155476)

[14.1. 근거자료(Source data) 21](#__RefHeading___Toc441155477)

[14.2. 증례기록서(Case report form) 22](#__RefHeading___Toc441155478)

[14.3. 자료 입력 과정(Data entry process) 22](#__RefHeading___Toc441155479)

[14.4. 연구 문서의 보관(Storage of study related documents) 22](#__RefHeading___Toc441155480)

[14.5. 자료의 질 보장(Quality assurance) 22](#__RefHeading___Toc441155481)

[14.6. 자료 보안(Confidentiality) 23](#__RefHeading___Toc441155482)

[**15.** **참고문서 (References)** 23](#__RefHeading___Toc441155483)

**연구의 명칭 및 실시기관(Study name and study center)**

**연구제목(Study name):** 건강 자원자에서 CES1 유전형에 의한 Oseltamivir 의 약동학적 특성을 평가하기 위한 임상연구(A clinical trial to investigate the influence of CES1 polymorphism on pharmacokinetic characteristics of oseltamivir in healthy Korean volunteers)

**실시기관(Study center):** 서울대학교 의과대학 임상약리학교실/서울대학교병원 임상약리학과(Department of Clinical Pharmacology and Therapeutics, Seoul National University School of Medicine and Hospital)

1. **임상시험실시기관명 및 주소(Name of study center and address)**

서울대학교병원 임상시험센터

서울특별시 종로구 대학로 101, 110-744

(Seoul National University Hospital Clinical trial center, 101 Daehak ro, Jong-no gu, Seoul, 110-744)

1. **임상시험책임자, 담당자 및 공동 연구자의 성명 및 직명(Name investigators)**
   1. **임상시험책임자(Principal Investigator)**

서울대학교 의과대학 임상약리학교실/ 서울대학교병원 임상약리학과

진료조교수 임 경 수 MD, PhD (Kyoung Soo Lim, MD, PhD)

- 1. **공동연구자(Co-investigator)**

서울대학교 의과대학 임상약리학교실/ 서울대학교병원 임상약리학과

부교수 유 경 상 MD, PhD (Kyung-Sang Yu, MD, PhD)

서울대학교 의과대학 임상약리학교실/ 서울대학교병원 임상약리학과

조교수 조 주 연 PhD (Joo-Youn Cho, PhD)

- 1. **임상시험 담당자(Sub-investigators)**

<별첨 2. 임상시험담당자 성명 및 직명 참조> (Described in the appendix 2)

- 1. **관리약사(Name of pharmacists)**

<별첨 3. 임상시험용의약품 관리약사의 및 직명 참조> (Described in the appendix 3)

1. **임상연구의 목적 및 배경(Background and purpose of study)**
   1. **배경(Background)**

Phase I 대사 단계 중 hydrolysis 반응에 esterase 대사효소가 관여하는 것으로 알려져 있으며 1990년대 이후 새로이 주목받고 있다. 이 중 carboxylesterase는 αβ hydrolase-fold family에 속하는 대사효소로써 esters, thioesters, carbamates, amids 등의 다양한 물질의 hydrolysis를 촉진시키는 역할을 한다. CES1은 주로 간에 분포하며 상대적으로 분자량이 작은 alcohol group과 분자량이 큰 acyl group을 가지는 cocaine, heroin, meperidine, demerol, lidocaine, lovastatin, methylphenidate, temocapril, oseltamivir 등의 약물이 기질 약물로써 대사작용을 나타낸다.

서양인에서 정신자극제인 methylphenidate의 약동학 연구를 수행하던 중 몇몇의 사람에서 약물 농도가 예측치보다 높게 측정되는 것이 확인되었다. 이로부터 CES1 gene에 관한 연구를 하게 되었으며 exon4 와 exon6의 coding region에서 single-nucleotide mutation을 확인할 수 있었다. Exon4에서의 p.Gly143Glu변이는 nonconservative substitution이며, exon6에서의 p.Asp260fs변이는 frameshift 변이이다. Oseltamivir는 전구 약물 (prodrug)로서, 경구 흡수된 oseltamivir가 인체 내에서 CES1 등에 의해 활성 물질인 Ro 64-0802 로 전환되어 (bioactivation) influenza virus neuraminidase 의 선택적 억제 효과를 지닌다. 최근 핀란드인 21명을 대상으로 한 임상시험 결과, CES1 gene의 polymorphism (p.Gly143Glu변이)이 oseltamivir의 bioactivation에 유의한 영향을 미치는 것으로 알려졌다. 반면에 일본인 30명을 대상으로 최근 시행된 임상시험 결과, CES1 gene의 polymorphism (CES1A diplotypes [a combination of haplotypes A (CES1A3-CES1A1), B (CES1A2-CES1A1), C (CES1A3-CES1A1 variant), D (CES1A2-CES1A1 variant)]가 oseltamivir의 약동학에 유의한 영향을 미치지는 않는 것으로 보고되었다. 그러나 이 연구에서는 1명의 대상자에서 최고혈중농도(Cmax) 및 혈중농도-시간곡선하 면적(AUC) 값이 다른 대상자들의 평균값의 10배 정도 높은 것으로 나타나 해석이 쉽지 않은 것으로 생각된다.

그러나, 한국인에서의 CES1 유전자변이에 따른 영향에 대한 연구는 아직 부족한 상황이다. 서울대학교 의과대학 임상약리학교실/서울대학교병원 임상약리학과에서는 이전부터 ‘약물유전체학 연구를 위한 한국인 자원자 혈액 검체 수집 연구’를 수행해왔다. 지난 2012년 5월부터 한국연구재단의 후원으로 이 중 200명의 혈액 검체를 대상으로 한국인에서의 CES1유전자의 유전형을 분석하여 다빈도 변이를 검출하는 연구를 진행하였고 그 결과 총 14개의 code가 nonsynonymous SNP로서 carboxylesterase 의 아미노산 변화를 유발함을 확인하였다. 또한 이 14개의 code를 대상으로 nonsynonymous SNPs (nsSNPs)에 대한 in silico 기능예측 프로그램인 Polyphen (http://genetics.bwh.harvard.edu/pph2)　프로그램을 사용하여 아미노산의 변화가 carboxylesterase 의 구조와 기능에 미치는 영향을 예측하였다. 분석 결과는 Probably damaging, possibly damaging, benign, unknown의 4가지 카테고리로 분류하였으며 그 중 인간 단백질 기능에 영향을 미칠 가능성이 큰 possibly damaging 이상의 카테고리에 해당하는 CES1 code 3개를 확인하였다. Probably damaging 카테고리에 CES1_23 code（56G>T, Amino Acid Change G19V, frequency: 1.5%）를 확인하였으며, possibly damaging 카테고리에 CES1_28 code (662A>G, Amino Acid Change E221G, frequency: 2.0%) 및 CES1_31 code (808G>T, Amino Acid Change A270S, frequency:０.８%) 를 확인하였다.

위 연구에서 기능변화 가능성이 예측되는 662A>G, E221G variant의 경우 한국인에서 2% 빈도로 발견된다. 이와 같은 변이가 있는 이들에선, oseltamivir의 bioactivation 이 저하되어, wild type에 비해 약물의 효능이 떨어질 것으로 예상된다. 비록 비교적 적은 빈도로 확인되는 변이라 할 수 있지만, 독감이 유행하는 경우 대다수의 국민이 oseltamivir를 복용할 가능성이 있어, 실제 그 영향을 받는 사람수가 상당히 많을 것으로 예상된다. 이에 이 유전변이가 enzymatic activity에 미치는 영향에 대해 건강 자원자대상 임상연구로 확인하고자 한다.

(Oseltamivir is one of the most commonly used antiviral agents to treat and prevent influenza. It is an ethyl ester prodrug of the active oseltamivir carboxylate, which selectively inhibits the neuraminidase enzyme of the influenza virus. Up to 80% of an orally administered dose of oseltamivir is converted into oseltamivir carboxylate, as mediated by human carboxylesterase 1 (CES1). The CES1 enzyme is expressed in most organs, particularly in the liver and intestine. Oseltamivir carboxylate is eliminated by urinary excretion. Other drug metabolizing enzymes (ie, cytochrome P450 or glucuronosyltransferases) are not involved in the elimination process of oseltamivir or its active metabolite.

The human CES enzyme is present as 2 main isozymes, ie, CES1 and CES2, and the level of expression of these CES isozymes differs among organs. Namely, the CES1 isozyme is highly expressed in the liver, whereas the expression of the CES2 enzyme is high in the intestine. Numerous ester prodrugs (eg, oseltamivir, clopidogrel, and angiotensin converting enzyme inhibitors), methylphenidate, and some illegal psychotropic drugs (eg, cocaine and heroin) are substrates for the CES1 enzyme.

Genetic polymorphisms of the human CES1 enzyme contribute to the large inter-individual variability of its substrate drugs. In earlier in vitro and in vivo studies, CES1 genetic variants such as c.428G>A (p.Gly143Glu, rs121912777) and c.780delT (p.Asp260fs, rs71647872) were associated with decreased activity of the CES1 enzyme, resulting in altered pharmacokinetic characteristics of methylphenidate, enalapril, clopidogrel and oseltamivir in humans. However, those CES1 genetic variants have not been observed in Asians and they are also rarely found in white, black and Hispanic populations. In Asian populations, the genetic polymorphism of CES1 enzyme was evaluated in Japanese subjects but the study results were inconsistent among the study design and the substrates. To find novel CES1 genetic variants which can alter the CES1 enzyme activity in Asian population, we identified 41 single nucleotide polymorphisms (SNPs) including 14 nonsynonymous variants for CES1 in 200 Koreans. Among them, 3 SNPs (ie, c.662A>G, rs200707504; c56G>T, rs3826190; c.808G>T, rs115629050) were predicted to decrease CES1 enzymatic activity based on an in silico analysis using the PolyPhen-2 software (http://genetics.bwh.harvard.edu/pph2/), and their minor allele frequencies (MAFs) in Koreans were 2%, 1.5% and 0.8%, respectively, compared to 4.57% in the global population. Whereas neither c56G>T nor c.808G>T was associated with a significant effect on CES1-mediated hydrolysis in a previous in vitro study, the effect of the c.662A>G SNP on the CES1 enzyme has not been characterized previously.

Many people may be exposed to oseltamivir during an influenza pandemic, and the c.662A>G SNP can be an important clinical biomarker if it significantly decreases CES1 enzyme function in humans, although the frequency of the c.662A>G SNP is relatively infrequent. Based on this understanding, we hypothesized that the CES1 c.662A>G SNP decreases the enzymatic activity of human CES1, thereby decreasing the bioactivation of oseltamivir. To test this hypothesis, the pharmacokinetics (PK) of oseltamivir and its active metabolite, oseltamivir carboxylate, were compared among 20 healthy male volunteers, classified into 2 genotype groups according to their c.662A>G variant status.)

- 1. **연구목적(Purpose of study)**

건강 성인을 대상으로 oseltamivir의 bioactivation에 영향을 미칠 것으로 예상되는 CES1 의 유전적 변이가 oseltamivir의 약동학에 미치는 영향을 평가한다. (To assess the effect of *CES1* genetic variability on the pharmacokinetics of oseltamivir)

1. **윤리적 고려사항(Ethical consideration)**

본 시험은 서울대학교병원 의학연구윤리심의위원회(IRB)의 승인과 헬싱키 선언 및 의약품 등의 안전에 관한 규칙[시행: 2013. 3. 23] 제 30조 및 별표 4의 의약품 임상시험 관리기준 등 관련규정을 준수하여 실시할 것이다. 이에 따라 시험의 목적 및 임상연구 의약품의 특성을 대상자에게 설명문을 통해 설명하며 <별첨 4. 동의서 및 대상자 동의 설명서 참조>, 시험의 목적 및 위험 등을 알고 동의서를 작성한 자원자만을 시험에 참여 시킬 것이며, 대상자는 시험기간 중 언제라도 본인이 원할 경우 시험 참여 동의를 철회할 수 있다는 것도 설명할 것이다. 시험기간 동안 얻어지는 결과들은 증례기록서에 기록될 것이며 모든 사항에 대해서는 비밀을 보장할 것이다. 보상에 관한 사항도 대상자에게 설명할 것이다. 이상반응 발생시 즉시 임상시험 담당자에게 보고하게 할 것이다. 필요한 경우 내원하여 검사 및 치료를 받을 수 있도록 하며, 증상이 소실될 때까지 추적조사 하도록 할 것이다. (This study will be performed after the approval of the Institutional Review Board of the Seoul National University Hospital, Seoul, Korea. This study will be conducted in accordance with the principles of the Declaration of Helsinki and ICH Good Clinical Practice. Written consent will be obtained from all the subjects before any study-related procedure are performed.)

1. **임상연구용 의약품(Study drug)**
   1. **시험약 Oseltamivir**
2. 제품명: 타미플루캅셀 75mg. ㈜한국로슈
3. 제형 및 성상: 백색 또는 미황색 가루가 든 하부 회색, 상부 미황색의 경질캅셀제
4. 함량: oseltamivir phosphate 98.5 mg (75 mg as oseltamivir)
5. 보관방법: 기밀용기, 25℃이하 보관
   1. **적응증(Indication)**
6. 1세 이상의 인플루엔자 A 또는 인플루엔자 B 바이러스 감염증의 치료
   (인플루엔자 감염의 초기증상 발현 48시간 이내에 투여를 시작해야 한다.)
7. 1세 이상의 인플루엔자 A 또는 인플루엔자 B 바이러스 감염증의 예방
   (인플루엔자 바이러스 감염증에 대한 예방의 일차요법은 백신요법이므로 백신에 당해 유행주가 포함되어 있지 않은 경우 또는 백신의 효과를 기대할 수 없거나 백신 접종을 하지 못하는 경우에 한하여 사용하며 이 약은 예방접종을 대체할 수 없다.)
   1. **용법, 용량, 예측 이상반응(부작용), 상호작용 및 사용상의 주의사항(Detailed information)**

<별첨1> 참조(Presented at appendix 1)

- 1. **임상연구용의약품의 공급 및 라벨링(Labeling of study drug)**

임상연구에 사용되는 의약품은 임상연구책임자가 시험약의 제조회사 등에 의뢰하여 공급을 받은 후, 임상시험센터 관리약사에게 공급한다.

임상시험용의약품의 포장 용기에는 다음과 같은 내용이 포함된 국문 라벨이 부착된다.

-  ‘임상 시험용’이라는 표시
-  제품의 코드명 또는 주성분의 일반명
-  제조 번호 및 사용(유효)기간 또는 재검사일자
-  저장 방법
-  임상시험계획 승인을 받은 자의 상호 및 주소
-  ‘임상시험용 외의 목적으로 사용할 수 없음’이라는 표시

Package box should be labeled as follows in accordance with Enforcement Regulation of Pharmaceutical Affairs Act Article 75 Paragraph 6 where investigational product labeling is stipulated.

1) Indication of “Investigational product”

2) Code name or generic name of the product

3) Lot number and expiration date

4) Storage condition

5) Name and address of the company who obtained the approval for the clinical trial

6) Indication of “Cannot be used for purposes other than clinical study”

1. **대상자 선정기준 및 대상자 수 산출 근거(Inclusion and exclusion criteria and determination of subject number)**
   1. **선정기준(Inclusion criteria)**
2. 모든 스크리닝 검사 이전에, 본 임상시험의 특성에 대해 설명을 들은 후, 본 시험의 참여에 자발적으로 동의하고 IRB 승인을 얻은 동의서에 서명한 자(Agreement with written informed consent)
3. 20세 이상 45세 이하의 건강한 한국인 남성 혹은 여성 자원자(Adult healthy male or female subject age 20 to 45)
   1. **제외기준(Exclusion criteria)**
4. 유의한 호흡기, 순환기, 신장, 위장관, 간, 내분비, 혈액, 신경 (운동실조 포함), 정신 질환 혹은 기타 만성 질환, 알코올 혹은 약물 중독에 대한 임상적인 증거가 있는 자 (Clinically significant, active gastrointestinal system, cardiovascular system, pulmonary system, renal system, endocrine system, blood system, digestive system, central nervous system, mental disease or malignancy
5. 임상연구용 의약품의 투약 14일 이내에 유의한 약물상호작용이 알려진 약물 또는 연구자가 판단하기에 적합하지 않다고 판단되는 약물을 복용한 자(Medication with any drug which may affect the pharmacokinetics of oseltamivir within 14 days)
6. 임상연구 개시 전 30일 이내 혈액을 공여하거나 60일 이내에 다른 임상시험용의약품 혹은 시판 중인 약물의 임상연구에 참여한 자(Previously donate whole blood within 30 days or Previously participated in other trial within 60 days)
7. 임상연구용 의약품에 대한 과민반응 등 유의한 이상반응을 보인 적이 있는 자(Subject with known for hypersensitivity reactions to oseltamivir)
8. 시험기간 중 임신을 계획하고 있거나 계획 중이 아니어도 인정받는 피임법 (예: 본인 및 파트너의 불임수술, 파트너의 자궁 내 피임기구, 간벽 피임법, 격막 또는 콘돔의 병용)을 사용할 수 없는 자(Subject who can not perform contraception during study periods)
9. 임신 또는 수유중인 여성 대상자(Female woman who are pregnant or are breast feeding)
10. 임상실험실검사 결과를 비롯한 기타 사유로 인하여 연구자가 임상연구 참여에 부적합하다고 판단한 자(An impossible one who participates in clinical trial by investigator's decision including laboratory test result)
    1. **대상자 수(Determination of subject number)**

본 연구는 탐색적 연구로서 대상자 산출을 위한 통계적 분석을 적용하지 않으며, 연구 목적을 충족시키는 한 가능한 최소의 대상자를 대상으로 하는 것이 바람직하다. 이와 같은 시험을 교차시험으로 설계 했을 때 집단간 비교를 가능케 하기 위해서는 군당 10 명의 대상자가 적당하며, 이번 시험은 단일 순서 시험으로 진행하므로 20명의 대상자를 하나의 군으로 진행한다. 한국인에서의 CES1 662A>G 변이가 있는 대상자 8 명과 wild type 대상자 12명의 약동학 파라미터를 비교함으로써 CES1 변이의 약물 노출에 대한 영향을 평가할 수 있을 것으로 예상하여 총 20명의 대상자를 모집한다.(The sample size was estimated to detect a 25% difference in the metabolic ratio of oseltamivir between the two genotype groups, with an 80% power and a 5% significance level. The total coefficient of variation value of the metabolic ratio in wild-type subjects was assumed to be 20% according to an earlier study)

1. **대상자의 관리(Restrictions to subjects)**

자원자에게 투약 7일전부터 연구 종료 시까지 과도한 운동을 하지 않고 투약 3일 전부터 음주나 카페인음료 섭취를 금하고 흡연을 하지 않으며 투약 14일전부터 일체의 타 약물의 복용을 하지 않도록 주의시킨다. (During the period from 7 days before hospitalization till end of study physical activities are restricted. During the period from 3 days before hospitalization till end of study alcohol consumption and smoking are restricted. During the period from 14 days before hospitalization till end of study any concomitant medication is restricted.)

1. **임상연구 기간(Study periods)**

IRB 승인일 이후 1년 또는 총 20명의 대상자가 임상연구를 완료하는 시점으로, 각 대상자는 첫 입원 후 10~12일 후에 임상연구를 종료한다. (1 year after IRB approval or until every subject complete the trial. Every subject completes the trial after 10 to 12 days after the drug administration)

1. **임상연구 방법(Study method)**
   1. **대상자 군 및 개요(Subject group)**

본 시험은 유전형에 따라 단회 입원, 단회 투약을 실시한다. 총 20 명의 대상자를 유전형에 따라 다음과 같이 모집한다.(A total of 20 subjects will be recruited according to the genotype test results)

|  | 662A>G | Wild type | 계(Total) |
| --- | --- | --- | --- |
| 대상자 수(Number of subject) | 8 | 12 | 20 |

- 1. **투약 방법 및 임상시험(Study drug admistration and study method)**

모든 투약은 임상연구담당자의 감독하에 실시된다

선정된 대상자는 입원 예정일(-1d) 오후 9시까지 서울대학교병원 임상시험센터에 입원한다. 입원 둘째 날(1d) 오전 9시경 Oseltamivir 75 mg capsule 을 물 240 mL와 함께 투약한다. 투약 직전 방광을 비운 후 48시간 동안 집뇨를 수행하며 예정된 시간에 약동학 채혈을 수행한다. (8mL씩 15회) 대상자는 48시간 째 약동학 채혈 후 퇴원할 수 있다.

모든 일정을 마친 대상자는 10d ~ 12d 중 하루에 post-study visit(PSV)을 한다.

(Subjects are admitted to the Clinical Trials Center at Seoul National University Hospital one day before the administration of oseltamivir. After an overnight fast, all the subjects will receive 75 mg of oseltamivir with 240 mL of water by the investigator. Subjects will perform post-study visit during day 10 – 12.)

Admission Drug administration Discharge Post-study visit


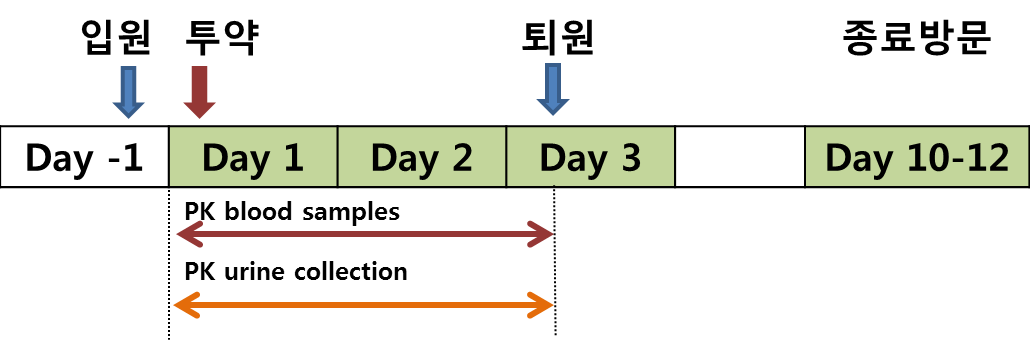


- 1. **채혈방법, 채혈량, 집뇨량 및 검체보관(Blood and urine sampling method, volume of blood and urine, sample handling method)**

약동학 평가를 위해 8 mL씩 각각 채혈한다.

또한, 입원 채혈 시 반복적 채혈을 위해 대상자의 팔 또는 손등의 정맥부위에 heparin-locked catheter를 설치하고. 채혈 시 채혈세트 안에 남아 있는 생리식염수를 제거하기 위해 매번 약 1 mL의 혈액을 빼내어 버린 후 혈액을 채취하고, 다시 catheter 안에 잔류하는 혈액의 응고를 방지하기 위하여 넣은 주사용 생리 식염수 1 mL를 주입한다.

약동학 평가를 위한 혈액은 헤파린이 포함된 채혈용기 (tube) 에 채취한 후 ice 용기에 담아 core lab으로 전달하여 30분 이내에 3000 rpm 으로 10분 동안 원심분리한 후, 상층액의 혈장만을 분리하여 3개의 eppendorf tube에 분주하여 분석시까지 -70℃이하에서 보관한다. 각 tube 에는 연구명, 대상자번호(AN), 채취시간 (약물 투여시간 기준) 등을 기록한 라벨을 부착한다.

또한 집뇨된 소변에서 구간별로 10 mL의 소변을 채취한 후 5개의 eppendorf tube에 1mL씩 분주하여 분석시까지 -70℃이하에서 보관한다. 각 tube 에는 연구명, 대상자번호(AN), 채취시간 (약물 투여시간 기준) 등을 기록한 라벨을 부착한다.

(Blood samples will be collected in following manners. A saline-locked angiocatheter will be inserted in the left or right forearm for blood sampling. At each sampling time, 8 mL of blood will be collected for PK sampling. Collected blood for the PK analysis will be put into a heparinized tube immediately, inverted gently to mix and centrifuged. Then, the plasma will be distributed into Eppendorf tubes as 3 aliquots, and frozen and stored at until dispatch for analysis.)

- 1. **순응도 평가(Assessment of compliance)**

연구 담당자가 직접 투약을 하며 복용을 확인한다.(Study drug will be administered by the investigator)

- 1. **채혈 및 집뇨 시간의 설정(Collection time for blood and urine samples)**

[약동학 검사]

시험약 단회 경구 투여 후 48시간까지 약동학적 평가를 위한 채혈과 집뇨가 이루어진다.

- 채혈시각
  - 투약직전, 투약 후 0.5, 1, 1.5, 2, 3, 4, 5, 6, 8, 10, 12, 24, 36, 48 h
- 집뇨 시각
  - 투약직전(10 mL), 투약 후 0-4 h, 4-8 h, 8-12 h, 12-24 h, 24-48 h

약동학 채혈 일정은 기존의 oseltamivir 임상 시험에 관한 참고문헌을 근거로 설정하였다.

허용시간
투약직전: 예정시각의 -30분

투약 후 0.5, 1, 1.5, 2, 3, 4, 5, 6, 8 h: 예정 시각의 ±3분

투약 후 10, 12, 24, 36, 48 h: 예정 시각의 ±5분

[안전성 검사]

안전성평가를 위한 채혈과 채뇨는 스크리닝 시와 시험 종료 시(PSV)에 실시한다.

(Serial plasma samples will be collected at 0 (ie, pre-dose), 0.5, 1, 1.5, 2, 3, 4, 5, 6, 8, 10, 12, 24, 36 and 48 h post-dose for the analysis of oseltamivir and oseltamivir carboxylate concentrations using a heparinized tube. Urine samples will also be collected up to 48 h post-dose.)

- 1. **대상자 SN 및 AN 부여 방법(Allocation of subject number)**

서면 동의를 받은 순서대로 자원자에게 스크리닝 번호(SN: Screening number)를 부여한다. 스크리닝 번호는 SN01로 시작하는 SN + 전체 숫자 두 자리로 구성된다. 재검까지 고려하여 최종적으로 스크리닝 통과가 결정되면 wild type 군은 AN001부터 AN012까지, variant 군은 AN101부터 AN108까지 전체 세자리 숫자로 구성되는 배정번호(AN: allocation number)를 부여한다. 본 시험은 맹검에 의해 영향을 받지 않는 약동학적 변수를 평가하므로 공개시험으로 진행되며, AN 배정 후 대상자가 투약 전 동의철회나 다른 이유로 인해 임상시험에 참여하지 못하는 경우 예비대상자를 두어 대체하는 것으로 한다. 중도 탈락 한 대체 대상자의 AN 번호는 각 군의 마지막 대상자번호 이후의 번호를 사용하도록 한다.

(Volunteers who agreed with written consent to participate in the study are assigned with a screening ID according to the time of consent. A screening ID consists of one letter 'S' and two digits, starting from 'SN01.' Study subject are assigned with a subject allocation number (AN). A wild type subject will receive AN001 to AN012 and the variant type subjects will receive AN101 to AN108. The screening and subject IDs assigned to each study subject are used as an identification number of the study subject through the end of the study.)

- 1. **검사 관찰 시점 및 항목(Assessment time and items)**
     1. **약물유전학 검사(Pharmacogenetic test)**
- 기존 연구(약물유전체학 연구를 위한 건강한 자원자 혈액 검체 수집, IRB no:H-0803-022-237)를 통해 임상약리학과에 보관된 유전자 검체 중 당시 시행한 연구 이외의 목적에 활용하는 것에 동의하였던 대상자들의 검체를 대상으로 약물유전학 검사를 시행한다.
- 검사대상 유전자 변이: Oseltamivir 약물반응에 관여하는 것으로서 CES1 662A>G 유전자변이에 대해 검사한다.
- Wild type의 경우 대부분의 한국인이 해당하므로, 유전자검사를 스크리닝 검사와 함께 시행 가능하다. 이 때 시행한 검사결과 variant type으로 나올 경우에도 연구에 참여 가능하다.
- 모든 피험자를 대상으로 스크리닝 시에 유전형 검사를 시행하여 유전형을 재확인한다. 3mL 가량의 혈액을 EDTA tube에 채혈 후 4°C에서 냉장 보관하여 24시간 내에 임상약리학과 유전자 분석 담당자에게 전달한다. (Genotype is evaluated with the banking whole blood samples of earlier study. The genotype of screened subject is re-evaluated with blood sample obtained at screening visit.)
  - 1. **스크리닝(Screening) 검사(D-30~D-1)(Screening visit. D-30 to D-1)**

스크리닝 방문을 하여 자발적으로 동의한 대상자에 한해 다음과 같은 항목들을 실시한다.

인구학적 정보, 문진, 신체검사, 임상실험실(혈액학, 혈액화학, 혈청, 뇨) 검사, 심전도, vital sign(At screening visit, following items are evaluated)

- 인구학적 정보 및 문진: 성별, 연령 등 인구학적 정보를 확인하고, 과거 병력, 최근 병력 및 약물 복용력 등을 문진한다. 또한 신장(소수점 3자리에서 반올림하여 2자리로 표기, m)과 체중(소수점 2자리에서 반올림하여 1자리로 표기, kg)을 측정하여 BMI를 계산한다.(Demographic data)
- 혈액학 검사(Complete blood test): WBC, RBC, Hemoglobin, Hematocrit, Platelet, differential count of WBC (Neutrophil, Lymphocyte, Monocyte, Eosinophil, Basophil)
- 혈액화학적 검사(Blood chemistry): Serum creatinine, Albumin, Total Bilirubin, SGOT, SGPT
- 뇨검사(Urine test): color, pH, specific gravity, albumin, bilirubin, glucose, urobilinogen, ketone, nitrite, occult blood, leukocyte, microscopy
- 혈청검사(Serology test): HBsAg, anti-HCV, anti-HIV
- 12-Lead 심전도(12-lead ECG)
- 여성 자원자의 경우 Urine HCG로 임신 여부 확인(Urine HCG test for woman subject)
  - 1. **투약 전/후(Before and after drug administration)**
- Vital sign: 1D (0, 3, 5)h, 3D 0h, 좌위 3분 휴식 후 SBP/DBP, HR 측정.
  허용 시간: 예정 시각의 ±10분
- 신체검사(Physical exam): 1D 0h: 투약 전 -30분 이내에 시행
  - 1. **종료방문 검사(Post-study visit)**
- Vital sign
- 혈액학 검사(Complete blood test): WBC, RBC, Hemoglobin, Hematocrit, Platelet, differential count of WBC (Neutrophil, Lymphocyte, Monocyte, Eosinophil, Basophil)

1. **대상자의 대체 및 탈락 기준(Subject dropout and replacement)**
   1. **분석군(Full Analysis set)**

투약 및 계획된 약동학 평가를 위한 채혈을 모두 마친 대상자를 시험의 완료자로 한다. 약동학 평가를 위한 채혈이 완전히 끝나지 않았거나 시험기간 중 다른 약물을 병용하는 등 유의한 계획서 이탈이 있는 경우 시험담당자의 판단에 따라 중도 탈락자로 한다. (Full analysis set is the most close and perfect group for analysis to which the intention-to-treat principle (ITT) that includes the entire study subject by randomized assignment can be applied. When a subject violates major screening criteria, misses at least one of the administrations or has no records after the randomized assignment, the subject is excluded from the full analysis set for analysis.)

- 1. **대상자의 대체(Subject replacement)**

시험을 중지하거나 시험에서 탈락되는 경우 새로운 대상자로 대체할 수 있다. 그러나 중지시점까지 해당 대상자로부터 얻은 시험결과는 최종 평가시 평가 가능한 항목에 대해서 검토될 수 있다. (A replacement of subject is considered when a subject withdraws the consent during the study. Results until the time of drop out from a subject can be examined when the study results can be evaluated at the final stage.)

- 1. **대상자의 탈락 기준(Dropout criteria)**

대상자의 시험탈락은 시험기간 중 어느 시점에서나 판정가능하며, 대상자 자의에 의해서 시험에서 탈락하는 경우에 그 사유는 불문하나 탈락 시에는 시험담당자 또는 책임자에게 보고토록 한다. 임상연구자는 다음과 같은 경우에 대상자를 시험에서 탈락시킬 수 있다.

- 대상자가 임상연구 중 시험약의 투여중단을 요구하거나, 시험참여 동의를 철회한 경우
- 중대한 이상반응/이상약물반응이 나타나는 경우
- 대상자가 이상반응이 발생하여 시험약의 복용을 거부하거나 더 이상 시험약의 복용이 힘들다고 판단되는 경우
- 대상자가 시험약의 안전성을 평가하는데 영향을 줄 것으로 예상되는 약물을 임의로 복용한 경우
- 시험자의 판단으로 환자를 위해 시험의 중지가 필요하다고 생각되는 경우
- 대상자가 제때에 방문을 안 하며 연락이 안 되는 경우

(‘Dropout’ means that an enrolled subject does not complete the study. Dropout could be decided at any time during the study period for the following reasons. Dropouts of enrolled subjects from the trial should be reported on the Clinical Report Form (CRF) with the date of the dropout, date of the last IP administration and the reason for the dropout.

- When the subject does not wish to proceed with IP administration or withdraws consent during the study period.

- In the cases where the subject requires to discontinue the investigational product or any case in which serious adverse event (SAE) or adverse drug reaction is observed

- The subject takes any medication that is expected to influence the safety and pharmacokinetic characteristics of investigational product, without permission of the investigator

- When the major protocol deviation occurs such as subject found not to be eligible based on inclusion and exclusion criteria after enrollment.

- In the cases where the principal investigator or sub-investigator decides to withdraw the subject from the clinical trial for other reasons)

1. **대상자의 안전 보호 및 이상반응 발생 등에 대한 대책**

- 임상연구 책임자 및 시험담당자는 연구계획을 정확히 분석, 숙지하여 예기치 않은 이상반응 출현에 대해 충분한 대처와 필요에 따른 보고, 참여 연구진에 대한 교육 등 사전 조치를 취한다. 임상연구의 진행은 의약품임상시험관리기준 (KGCP)에 합당하게 진행한다.
- 본 연구 도중 부작용이 발생하는 경우에는 적절한 조치를 취한다. 즉, 중대하고 예기치 못한 이상약물반응의 발생시 IRB에 보고한다. 이상반응 등에 대한 필요한 검사 및 치료는 의학적으로 관행적인 요법에 준하여 시행한다.
- 응급상황 발생시 서울대학교병원 및 임상시험센터 응급상황에 대한 대처방안에 준하여 조처한다.

(This study will be conducted in accordance with Korea Good Clinical Practice(KGCP) and the principles of ‘The Declaration of Helsinki’. ICH Guideline E2A ‘Clinical Safety Data Management: Definitions and Standards for Expedited Reporting’ will also be complied with.)

1. **평가방법(Endpoints)**

약동학 평가와 통계분석은 모든 채혈 일정을 완료한 자를 대상으로 한다. 안전성 평가는 투약 받은 모든 대상자를 대상으로 한다.(Pharmacokinetic analysis will be performed using full analysis set.)

- 1. **약동학 평가(Pharmacokinetic assessment)**

**평가 변수: (Pharmacokinetic parameters)**Oseltamivir 및 active metabolite (oseltamivir carboxylate)의 C_max_, AUC_last_, AUC_inf_, CL/F, CL_R_/F, t_1/2_, metabolic ratio 등

위의 지표들에 대하여 비구획적 방법 또는 집단 비선형혼합효과 모델링(NONMEM)을 이용하여 약동학 파라미터를 산출할 수 있다. 산출된 파라미터에 대한 유전형별 공변량 효과 분석 및 정량화를 실시한다. (Cmax, AUClast, AUCinf, CL/F, CLR/F, t1/2, metabolic ratio of oseltamivir and its active metabolite)

- 1. **통계분석(Statistical analysis)**
- Oseltamivir 및 active metabolite (oseltamivir carboxylate)의 약동학 파라미터(C_max_, AUC_last_, AUC_inf_, CL/F, CL_R_/F, t_1/2_, metabolic ratio)는 비구획방법을 이용하여 산출한다.
- 약동학적 파라미터들을 유전형별로 기술통계학적으로 분석한다.
- Wild type에 대한 662A>G 군의 약동학 파라미터들(C_max_, AUC_last_, CL/F, metabolic ratio)에 대한 점추정치를 구하고 유의수준 0.05에서 이의 90% 신뢰구간을 산출한다.
- 필요시 기타 적합한 통계분석 방법을 이용하여 분석할 수 있다.

(The plasma concentrations of oseltamivir and oseltamivir carboxylate are analysed by noncompartmental analysis using Phoenix® WinNonlin® software version 1.3 (Certara, St. Louis, MO, USA). All the demographic characteristics and PK parameters will be presented as arithmetic mean and standard deviation (SD). A general linear model was developed to estimate the geometric mean ratios (GMRs) of the PK parameters and their 90% confidence intervals (CIs) for heterozygous carriers (c.662AG) over non-carriers (c.662AA). The Wilcoxon two-sample test will be performed to identify significant differences in the demographic characteristics between the genotype groups. A p-value <0.05 is considered statistically significant. Statistical analyses will be performed using SAS software version 9.3 (SAS Institute Inc., Cary, NC, USA).)

- 1. **안전성 평가(Safety assessment)**
- 임상검사: 필요시 정량적인 안전성 및 내약성 자료에 대해 기술통계학적으로 제시할 수 있다.
- 병력과 이상반응은 MedDRA (버전 12.0 또는 사용 가능한 최신 버전) 용어로 기술한다.

(Adverse events data including number of events, number of subjects, intensity, seriousness, and causality can be analyzed by genotype group.)

1. **이상반응(Adverse event, AE)**
   1. **이상반응(Adverse Event, AE)의 정의(Definition of AE)**

임상연구에 사용되는 의약품을 투여 받은 대상자에서 발생한, 바람직하지 않고 의도되지 않은 증후(sign, 예: 실험실적 검사치의 이상), 증상(symptom), 질병을 말하며, 해당 임상연구에 사용된 의약품과 반드시 인과관계를 가져야 하는 것은 아니다. (An adverse event (AE) is defined by the ICH-GCP Guidelines as [any untoward medical occurrence in a clinical study subject administered a medicinal product], and does not necessarily have a causal relationship with the medicinal product.)

- 1. **이상약물반응(Adverse Drug Reaction, ADR)의 정의(Definition of adverse drug reaction)**

임상연구에 사용되는 의약품의 임의의 용량에서 발생한, 모든 유해하고 의도되지 않은 반응으로서, 임상연구에 사용되는 의약품과의 인과관계를 배제할 수 없는 경우이다.(An adverse drug reaction is defined as AE related with study drug administration.)

- 1. **중대한 이상반응/이상약물반응(Serious AE/ADR)의 정의(Definition of serious AE/ADR)**

임상연구에 사용되는 의약품의 임의의 용량에서 발생한 이상반응 또는 이상약물반응 중에서 다음과 같은 경우를 말한다.

- 사망을 초래하거나 생명을 위협하는 경우
- 입원 또는 입원기간의 연장이 필요한 경우
- 지속적 또는 의미 있는 불구나 기능 저하를 초래하는 경우
- 선천적 기형 또는 이상을 초래하는 경우
- 기타 의학적으로 중요한 상황

(A serious adverse event (experience) or reaction is any untoward medical occurrence that:

- resulted in death or is life-threatening,
- required inpatient hospitalization or prolongation of existing hospitalization,
- resulted in persistent or significant disability/incapacity,
- resulted in a congenital anomaly/birth defect,
- is of other medical significance.)
  1. **이상반응의 기록(Recording of adverse event)**

이상반응은 의학 진단 용어로 기록하여야 하며, 이것이 불가능할 경우 임상연구책임자 또는 담당자가 관찰하거나, 대상자가 보고한 증상 및 징후에 대한 용어를 기록하여야 한다.

동의서 취득 전부터 대상자에게 관찰되었던 증상 및 징후는 대상자의 증례기록서에 기록하여야 한다. 동의서 취득 후, 임상연구용 의약품의 투약 전에 나타난 이상반응은 관련 근거문서 (예, screening 문서, worksheet 등)에 기록하여야 한다. 시험약 투약 후 나타난 모든 이상반응은 약물과 인과관계가 없더라도 CRF의 이상반응 기록지에 기록하여야 한다.

임상연구 도중 또는 최종투약 후 30일 이내에 나타나는 중대한 이상반응은 이상반응의 시험약과의 관련 여부와 관계없이 임상연구책임자 또는 담당자는 24시간 이내에 IRB에 보고하여야 한다. 이상반응은 시험책임자 또는 담당자가 평가한다. 처음 기록되는 이상반응은 CRF의 이상반응 모니터링 기록서에 기록한다. CRF에는 이상반응의 증상 및 증후, 시험약과 관련하여 취해진 조치, 발현 날짜 및 시간(가능한 경우), 중증도(최대 강도; maximal intensity), 경과(course; 즉, 지속적 또는 간헐적), 이상반응의 중대성(seriousness), 결과(outcome), 시험약과의 인과관계를 기재하여야 한다. 세부적인 용량 변경 및 치료 내용은 CRF의 적절한 page에 기록하여야 한다.

시험약 투약 후 나타난 이상반응 중 종료 방문 이후에도 지속되는 이상반응은 필요한 경우 이후 방문에서 검토되어야 한다. 이상반응이 회복된 경우에는 CRF의 기록이 완성되어야 한다.

이상반응을 알아내는 한 가지 일관된 방법으로서 대상자의 자발적인 보고 외에도, 투약시 마다 대상자에게 다음과 같이 원하는 대답을 유도하지 않는 방식으로 질문을 한다. “지난 방문 또는 투약 이후로 다르게 느껴진 바는 없습니까?”

(The investigator will confirm the medical status of the subjects through inquiries and examinations. All the adverse events including status changes after enrollment will be recorded. Adverse events will be confirmed by the inquiries of the investigator or by voluntary reporting from the subjects, and will be recorded in terms of symptoms and signs, duration (onset and termination date/time), intensity (mild, moderate, and severe), causal relationship to study drug, action taken, seriousness, etc. AE terms will be recorded using MedDRA®.)

- 1. **이상반응의 중증도 및 시험약과의 인과관계 평가(Assessment method for severity and relationship of AE with IP)**
     1. **이상반응의 중증도 평가(Assessment method for severity of AE)**

이상반응의 중증도는 최대 강도(maximal intensity)에 의거하여 아래의 기준에 의해 분류한다.

1) 경증 (mild): 대상자의 정상적인 일상생활(기능)을 저해치 않고, 최소한의 불편을 야기하여, 대상자가 쉽게 견딜 수 있는 경우

2) 중등증 (moderate): 대상자의 정상적인 일상생활(기능)을 유의하게 저해하는 불편을 야기하는 경우

3) 중증 (severe): 대상자의 정상적인 일상생활(기능)을 불가능하게 하는 경우

The maximum intensity of AE is categorized by grade defined as below.

1 (Mild) Awareness of signs or symptoms, but easily tolerated; is of minor irritant; causing no loss of time from normal activities

2 (Moderate) Discomfort severe enough to cause interference with usual activities

3 (Severe) Incapacitating with inability to work or do usual activity

- - 1. **이상반응의 시험약과의 인과관계 평가(Assessment method for relationship of AE with IP)**

임상연구용 의약품과의 인과관계는 다음과 같이 6단계로 분류하고, 시험담당자의 견해를 부가한다.

1) 명확히 관련성 있음 (Definite)

2) 관련성이 있다고 생각됨 (Probable)

3) 관련 가능성이 있음 (Possible)

4) 관련성이 없다고 생각됨 (Unlikely)

5) 명확히 관련성 없음 (Not related)

6) 알 수 없음 (Unassessable)

(The principal investigator or study staff who is a physician will assess the causal relationship between an AE and study drug (or placebo) considering the knowledge on the subject, circumstances around the event, and all other causes that can be thought of.)

- - 1. **이상반응의 추적 관찰(Follow up of AE)**

임상연구 책임자 또는 담당자는 이상반응이 나타난 대상자에 대해 증상이 가라앉고 비정상적 임상검사치가 기준치로 회복되거나, 혹은 관찰된 변화에 대해 만족스러운 설명이 될 때까지 추적 관찰하여야 한다. (The investigators will follow up AE’s until every AE are disappear)

- 1. **중대한 이상반응의 보고(Reporting serious AE)**

중대한 이상반응이 임상연구용의약품 투여기간 도중 또는 최종 투약 후 30 일 이내에 발생하는 경우, 이상반응의 시험약과의 관련 여부와 관계없이 임상연구책임자 또는 담당자는 24시간 이내에 IRB에 보고하여야 한다.

(In case of a serious adverse event during drug administration, the principal investigator is to report the fact to the IRB within 24 hours or by the next working day the latest, by telephone or fax.)

1. **자료 처리 및 자료의 질 보장(Data management and quality assurance)**
   1. **근거자료(Source data)**

근거자료는 의무기록, 심전도, 임상실험실 검사 결과, 약 불출 기록 등의 모든 문서, 자료, 기록을 의미한다. 연구 기간 중에 모아진 자료들은 적절한 근거자료에 기록되어야 한다.

스크리닝/등록 로그에 기록된 자료에는 대상자 인식번호, 스크리닝 날짜, 탈락 사유(탈락시) 등이 기록되어야 한다. 스크리닝 된 모든 자원자들은 이 로그에 기록되어야 한다.

연구 모니터링, 점검, IRB 검토, 규제기관의 실태조사 시 근거자료에 대한 열람이 허락된다.

(Source data refers to all documentations, data, records such include but not limited to Electronical Medical Record/ Chart, ECG, Laboratory tests, study drug accountability records. Informations collected during the clinical study will be recorded in appropriate source documentations. The information recorded in screening/enrollment log should include screening number, screening date, drop-out reasons (if dropped-out). All screened subjects should be listed in this screening/enrollment log.Direct access to this source data is allowed if study-related monitoring, audit, IRB review, inspections from local regulatory authority is required.)

- 1. **증례기록서(Case report form)**

증례기록서에 기록된 모든 정보는 대상자의 근거문서에 바탕한다.

등록된 모든 대상자의 증례기록서가 완성되어야 한다. 모든 증례기록서는 검정색 볼펜으로 명확하게 기록하여야 한다. 수정이 필요한 경우에는 틀린 곳을 한 줄로 긋고, 수정된 내용과 수정자의 서명과 수정 날짜를 기록하도록 한다.

임상시험책임자는 증례기록서의 완성여부와 정확도를 검토한 후, 일시를 기록하고 서명한다.

(All records on the case report forms (CRF) will be based on the subject source document.

CRF for subjects who provided the informed consent must be complete.)

- 1. **자료 입력 과정(Data entry process)**

증례기록서에 기록된 자료는 Promasys® 을 이용하여 개발된 데이터베이스 시스템에 입력된다. 자료 입력 과정은 임상시험기관의 SOP 에 따라 이루어진다. (Data entry will be implemented according to SOP of the study site.)

- 1. **연구 문서의 보관(Storage of study related documents)**

필수 보관 문서를 임상시험 종료일로부터 3년간 보존한다. (The investigator will be responsible for retaining all records pertaining to the study (including CRF, informed consent form, clinical study report and other related records) for 3 years from the end of study.)

- 1. **자료의 질 보장(Quality assurance)**

임상 시험 시작 전에 모든 연구자가 참여한 개시모임을 개최한다. 이 모임에서 연구계획서, 연구 수행, 증례기록서 완성, 샘플 수집 및 전처리 방법 등에 관한 자세한 논의를 한다. 임상시험이 KGCP 에 따라 실시되도록 자체 모니터링을 실시한다. 모니터링 시에는 증례기록서에 기록된 모든 자료를 이중 확인 하도록 하며, 이상이 발견되면 근거문서와 비교 검토한다. 증례기록서의 자료를 데이터베이스 시스템에 이중 입력 방법을 통하여 입력하도록 하며, 입력된 자료를 시험기관의 SOP 에 따라 이중 확인하도록 한다. 자료 입력 완료가 확인되면, 데이터베이스 시스템을 lock 한다. (Investigator will hold an initiative meeting. The detailed discussion on study protocol, study procedure CRF entry and completion guideline, collection of samples and handling will be done in this meeting. A monitor from Seoul National University Hospital Clinical trial center will monitor as specified in the monitoring plan and verify the CRF based on all source documentations. If decrepancy is observed, the data should be verified with source data. Data entered in CRF will be enterd into database system. Double data entry will be implemented according to the study site SOP. Once all data entry is completed and confirmed, database system lock will be done.)

- 1. **자료 보안(Confidentiality)**

연구를 통하여 얻어진 근거문서, 증례기록서 등의 모든 문서에 대해서는 보안이 유지되어야 한다. 대상자의 익명성이 보장되어야 하므로, 모든 문서에 대상자 이름 대신 대상자 번호나 이니셜을 사용한다. 대상자를 식별할 수 있는 문서도 연구자에 의해 보안이 유지되어야 한다.

(All information obtained during the conduct of the study which relates to an individual volunteer will be regarded as confidential. Throughout the study documentation subjects will be referred to by the number allotted to them and will not be referred to by name in any document concerning the study disclosed to any person not under the direct control of the investigator.)

1. **참고문서 (References)**

1. Nemoda, Z., et al., *Carboxylesterase 1 gene polymorphism and methylphenidate response in ADHD.* Neuropharmacology, 2009. **57**(7-8): p. 731-3.

2. Tarkiainen, E.K., et al., *Carboxylesterase 1 polymorphism impairs oseltamivir bioactivation in humans.* Clin Pharmacol Ther, 2012. **92**(1): p. 68-71.

3. Suzaki, Y., et al., *The effect of carboxylesterase 1 (CES1) polymorphisms on the pharmacokinetics of oseltamivir in humans.* Eur J Clin Pharmacol, 2013. **69**(1): p. 21-30.

4. Davies, B.E., *Pharmacokinetics of oseltamivir: an oral antiviral for the treatment and prophylaxis of influenza in diverse populations.* J Antimicrob Chemother, 2010. **65 Suppl 2**: p. ii5-ii10.

5. Wattanagoon, Y., et al., *Pharmacokinetics of high-dose oseltamivir in healthy volunteers.* Antimicrob Agents Chemother, 2009. **53**(3): p. 945-52.

6. He, G., J. Massarella, and P. Ward, *Clinical pharmacokinetics of the prodrug oseltamivir and its active metabolite Ro 64-0802.* Clin Pharmacokinet, 1999. **37**(6): p. 471-84.

1. CES 유전형 검사 (채혈량 3 mL, 약물유전체학 연구를 위한 건강한 자원자 혈액 검체 수집, IRB no:H-0803-022-237 을 통해 유전형이 확인된 자원자의 경우에도 유전형 재확인을 위해 시행). (Genotype is evaluated with the banking whole blood samples of earlier study) [↑](#footnote-ref-2)
2. 약동학 채혈: 투약직전, 투약 후 0.5, 1, 1.5, 2, 3, 4, 5, 6, 8, 10, 12, 24, 36, 48 h (Serial plasma samples will be collected at 0 (ie, pre-dose), 0.5, 1, 1.5, 2, 3, 4, 5, 6, 8, 10, 12, 24, 36 and 48 h post-dose) [↑](#footnote-ref-3)
3. 집뇨: Oseltamivir 투약직전 방광을 비우고(이때 10 mL 가량의 검체 수집) 복용 후 다음의 시간대 별 집뇨를 시행한다. 0-4 h, 4-8 h, 8-12 h, 12-24 h, 24-48 h (Urine samples will be collected up to 48 h post-dose) [↑](#footnote-ref-4)
4. 임상실험실 검사: 스크리닝, PSV에 시행 (스크리닝: 혈청, 혈액학, 혈액화학, 소변, 여성 피험자는 urine HCG 추가 / PSV: 혈액학) (Laboratory test: CBC, blood chemistry, urine test. Urine HCG for woman) [↑](#footnote-ref-5)
5. 12-lead ECG: 스크리닝 시에 시행(Performed at screening) [↑](#footnote-ref-6)
6. Vital sign(SBP/DBP, HR): 스크리닝, 1d: (0, 3, 5)h, 3d 0h, PSV에 시행 (Performed at screening, 1d: (0, 3, 5)h, 3d 0h and PSV) [↑](#footnote-ref-7)
7. 신체검사: 스크리닝, 1d 0h에 시행 (Physical exam is performed at screening and 1d 0h)

   *이상반응 모니터링은 입원시부터 종료방문시까지 연속하여 시행한다. (Adverse event is monitored during entire study periods) [↑](#footnote-ref-8)
